# Sustainable production of insecticidal and acaricidal metabolites by endophytic fungi using solid-state fermentation

**DOI:** 10.1038/s41598-026-40413-w

**Published:** 2026-04-03

**Authors:** Mervat Morsy Abbas Ahmed El-Gendy, Hanaa E. Sadek, Marwa E. Barghout, Samar S. Ibrahim, Huda H. Elbehery, Ahmed Mohamed El-Bondkly

**Affiliations:** 1 Chemistry of Natural and Microbial Products Department, National Research Centre, El- Buhouth Street, Dokki, Giza, 12622 Egypt; 2 Pests and Plant Protection Department, National Research Centre, El-Buhouth Street, Dokki, Giza, 12622 Egypt; 3 Genetics and Cytology Department, National Research Centre, El- Buhouth Street, Dokki, Giza, 12622 Egypt

**Keywords:** *Agrotis ipsilon*, Biological control, Endophytic *Geomyces* sp., *Tetranychus urticae*, *Triticum aestivum*, Biochemistry, Biological techniques, Biotechnology, Microbiology, Plant sciences

## Abstract

The purpose of this study was to isolate and assess endophytic fungi from wheat plants as potential biocontrol agents against major wheat pests. A total of 38 fungal isolates, obtained from the Delta, Middle, and Upper regions of Egypt, were examined for their ability to produce bioactive metabolites exhibiting insecticidal and acaricidal activities. Among them, isolate MORSY-27 exhibited remarkable biosynthetic potential, yielding notably high levels of cuticle-degrading enzymes. It also synthesized substantial amounts of phytochemicals, including alkaloids, phenolics, terpenoids, flavonoids, and fatty acids. According to morphological, biochemical, and molecular analyses, the isolate was identified as *Geomyces *sp. MORSY-27. Different agro-industrial residues were evaluated as cost-effective substrates for solid-state fermentation to reduce production expenses; among these, sunflower cake supported the highest metabolite production. Extracts derived from *Geomyces *sp. MORSY-27 exhibited strong insecticidal activity against *Agrotis ipsilon*, significantly impairing larval and pupal development. Additionally, the extracts exhibited potent, concentration- and time-dependent acaricidal effects, achieving nearly 100% adult mortality of *Tetranychus urticae* and complete inhibition of egg hatching. Gas chromatography-mass spectrometry analysis revealed the presence of 39 bioactive compounds. These findings indicate that *Geomyces *sp. MORSY-27 demonstrates significant potential as a sustainable and cost-effective source of natural biopesticides, offering valuable potential for integrated pest management.

**Supplementary Information:**

The online version contains supplementary material available at 10.1038/s41598-026-40413-w.

## Introduction

 Wheat (*Triticum aestivum* L.) is cultivated worldwide under a wide range of environmental conditions and serves multiple purposes, including use as food, feed, fodder, and industrial raw material. This versatility offers stakeholders vast opportunities to diversify production, enhance value addition, and generate employment^1^. However, wheat productivity is adversely affected by biotic factors (i.e., pests and pathogens) and abiotic stresses associated with environmental challenges. Among these, insect pests pose a significant biotic constraint to global wheat production, resulting in substantial yield and quality losses^2^.

Major wheat pests, including aphids, stem borers, Hessian flies, black cutworms, and wheat midges, attack various parts of the plant organs, such as leaves, stems, roots, and developing grains, leading to stunted growth, reduced photosynthesis, and impaired nutrient uptake^3^. The extent of damage depends on pest type, infestation level, crop growth stage, and environmental conditions; in severe outbreaks, yield losses can reach up to 100%, posing serious economic and food security challenges for wheat-dependent populations^4^. Among the diverse species in agricultural ecosystems, the family Noctuidae is the most species-rich in the order Lepidoptera^5^. Among these pests, the extremely damaging black cutworm, *Agrotis ipsilon* (Lepidoptera: Noctuidae), poses a major threat to a wide range of crops, particularly during the seedling stage^6^. Female *A. ipsilon* lay approximately 2,000 eggs on the roots, stems, and low-lying vegetation of the host plant, with newly hatched larvae initially feeding on leaves before progressing to stems^7^. This species attacks cultivated plants from at least 15 families, including important crops such as wheat, rice, maize, potato, tomato, cotton, pea, okra, cabbage, cauliflower, and coffee^8^. The larvae are highly voracious, feeding aggressively on leaf buds and stems, and can potentially destroy entire plants if left uncontrolled. Consequently, effective management of *A. ipsilon* is critical for safeguarding crop yields and supporting the agricultural sector^7^.

The two-spotted spider mite, *Tetranychus urticae* Koch (Acari: Tetranychidae), is one of the most widespread and destructive arthropod pests affecting global agriculture^9^. It is considered a constant threat to agriculture due to its exceptional capacity to develop pesticide resistance, its short life cycle, high reproductive capacity, and its broad polyphagy^10^. This mite infests over 1,100 plant species across numerous families, including vegetables, fruits, and ornamental crops^11^. Although *T. urticae* is typically associated with horticultural crops, it has also been documented feeding on cereal crops, such as wheat, under favorable environmental conditions^12^. Infestations are particularly prevalent in arid and semi-arid regions, where elevated temperatures and low humidity facilitate rapid population expansion^13^. Due to its broad host range, high fecundity, and adaptive ability, *T. urticae* remains one of the most challenging pests to manage.

A range of strategies is used to manage agricultural pests, including chemical insecticides, resistant crop varieties, and biological control agents. However, the extensive application of chemical pesticides has raised significant concerns regarding toxicity, environmental contamination, and the development of pest resistance. The need for sustainable, biologically based pest management solutions has been highlighted by their detrimental impacts on beneficial organisms, including microorganisms, pollinators, and natural predators, as well as on humans, animals, and plants^2^. Among these alternatives, entomopathogenic fungi, particularly species of *Beauveria* and *Trichoderma*, are among the most widely studied and applied biological control agents for managing insect pests worldwide. Their ability to infect and suppress pests is largely attributed to their production of lytic enzymes, including lipase, protease, and chitinase^14,15^.

Endophytic fungi are recognized as prolific producers of structurally diverse secondary metabolites with strong pesticidal potential. Their natural adaptation to plant tissues enables them to synthesize bioactive compounds that contribute to plant defense mechanisms^16^. When coupled with solid-state fermentation (SSF), these fungi can be exploited more efficiently, as SSF closely resembles their natural growth environment and generally enhances secondary metabolite yields compared to submerged systems^17^. Additionally, SSF offers notable sustainability advantages by utilizing low-cost agro-industrial residues as substrates, minimizing water and energy use, and allowing cost-effective, scalable production of fungal pesticidal metabolites^18–20^.

Endophytic fungi establish symbiotic relationships within plant tissues without causing visible harm, and their interactions play a vital role in enhancing the survival of both partners. They provide substantial benefits to their host plants, particularly under biotic and abiotic stress conditions^21^. Endophytic microbes protect plants from pest attacks through multiple mechanisms, including the production of phytochemicals (such as phenolics, flavonoids, terpenoids, alkaloids, steroids, coumarins, benzopyranones, and quinones) and other bioactive secondary metabolites. These compounds, ranging from volatile organic compounds (VOCs) and lipopeptides to cuticle-degrading enzymes, exhibit diverse chemical structures and hold considerable potential for agricultural applications^15^.

Entomopathogenic fungi, especially *Beauveria *spp., are well known for their wide range of insect hosts and for well-defined infection mechanisms that include toxin synthesis, hydrolytic enzymes, and cuticle penetration^22^. They are widely used commercially in biological control since their effectiveness has been well confirmed in both laboratory and field settings^23^. In contrast, *Geomyces *spp. are mostly psychrotolerant fungi commonly found in moderate and cold climates^24^. Despite these fungi being known to exhibit a variety of metabolic and extracellular enzymatic activities characteristic of psychrophilic fungi, their potential as entomopathogens, including their host range and infection processes, still needs further study^25^.

Therefore, this study aimed, for the first time, to: (1) isolate endophytic fungi from healthy wheat roots and evaluate their ability to produce a range of bioactive metabolites under solid-state fermentation, including cuticle-degrading enzymes (lipase, protease, and chitinase), antioxidant enzymes (superoxide dismutase and peroxidase), phytochemicals (phenolic, flavonoids, terpenoid, and alkaloid compounds), and fatty acids; (2) assess, for the first time, the potential of *Geomyces* as a biocontrol fungus by utilizing six agro-industrial residues as low-cost substrates for production against *A. ipsilon* and *T. urticae*; and (3) identify the volatile organic compounds of the most effective extract using gas chromatography-mass spectrometry (GC-MS) analysis.

## Materials and methods

### Chemicals and substances

All chemicals were supplied by Sigma-Aldrich Chemical Co. (St. Louis, MO) and Merck (Darmstadt, Germany). Six types of agro-industrial residues, orange pomace (OP), taro pomace (TP), banana pomace (BP), sunflower cake (SFC), tomato pomace (ToP), and molokhia stalk (MS), were collected from local suppliers. The residues were washed, dried, ground, and sieved to 0.5 mm, then used as a powder.

### Sampling site and isolation of endophytic fungi

Endophytic fungal isolates were obtained from the roots of healthy, symptom-free wheat plants collected from three locations in Egypt: Abu Ḥammad, Sharqia Governorate in the Nile Delta (30.7°N 31.63°E); Matai, El-Minia Governorate in Middle Egypt (28°07′10″N 30°44′40″E); and Girga, Sohag Governorate in Upper Egypt (26°33′N 31°42′E) (Fig. S1a-d). Samples were placed in sterile plastic bags on February 1 st, transported to the laboratory in an ice tank, and stored at 4 °C until further processing.

Endophytic fungi were isolated from mature, healthy, and symptomless wheat roots following a standard surface-sterilization procedure^26,27^. Plant roots were first thoroughly rinsed under tap water to remove adhering soil and debris, then immersed in 70% ethanol for 2 min and washed three times with sterile distilled water. This was followed by immersion in 2.5% sodium hypochlorite (w/v) for 2 min, another rinse with sterile distilled water, immersion in 70% ethanol for 1 min, and a final rinse with sterile deionized water to eliminate any residual ethanol or sodium hypochlorite. The sterilized root segments were dried on sterile filter paper and cut into 0.5 cm pieces. These segments were then aseptically placed on potato dextrose agar (PDA) supplemented with 10% wheat powder (autoclaved) plates to stimulate the growth of endophytic fungi. To verify the effectiveness of the surface-sterilization process, an aliquot of the sterile water from the final rinse was plated onto fresh PDA as a control. All plates were incubated in the dark at 25 ± 2 °C for 10 days until fungal colonies emerged from the root tissues. Emerging hyphal tips were subcultured onto fresh PDA plates and transferred repeatedly until pure isolates were obtained.

### Growth conditions and extraction of bioactive metabolites from endophytic fungi

Ten grams of taro pomace (TP), adjusted to 55% moisture content at pH 5.5, were placed in 250 mL Erlenmeyer flasks, autoclaved, and inoculated with two 2-cm-diameter discs from a 7-day-old culture of each tested fungal strain. The flasks were incubated individually for 10 days in the dark at 25 °C under static conditions, in duplicate for each of two experimental groups. The first group was used for the extraction of extracellular enzymes: the fermented taro pomace was mixed with 100 mM citrate-phosphate buffer (pH 6.0) at a 1:4 w/v ratio, agitated on a rotary shaker at 150 rpm and 30 °C for 60 min, then filtered through Whatman No. 1 filter paper using a Büchner funnel. The resulting clear supernatants were collected and used as enzymatic crude extracts (ECE). The second group was used to extract phytochemical metabolites with ethanol (5:1 w/v, performed twice) under agitation at 150 rpm for 3 h. The resulting mixture was filtered through a Whatman No. 1 filter paper using a Büchner funnel and then concentrated to dryness under vacuum in a rotary evaporator. For further analysis, each crude extract was freshly dissolved in 10% dimethyl sulfoxide (DMSO) to the desired concentrations. The extracts were stored at −20 °C to prevent degradation until use in screening and optimization studies of cuticle-degrading enzymes, phytochemicals, antioxidant enzymes, and pesticidal activity.

### Enzymatic activity assays

The fungal endophytes were evaluated for their ability to produce extracellular cuticle-degrading enzymes, including extracellular chitinases, lipases, and proteases, as well as antioxidant enzymes, such as superoxide dismutase and glutathione peroxidase. All assays were conducted in triplicate, and enzyme activities were expressed as U/gds (grams of dry substrate).

#### Lipase activity

Lipase activity in the enzymatic crude extracts (ECE) was determined following the method of Beys da Silva et al.^28^. Briefly, 20 µL of ECE was mixed with 230 µL of substrate solution, which was prepared by dissolving 3 mg of *p*-nitrophenyl palmitate (*p*NPP) in 1 mL of isopropanol and adding it to 9 mL of 50 mM Tris–HCl buffer (pH 8) containing 1 mg/mL Arabic gum and 4 mg/mL Triton X-100. The reaction mixture was incubated at 37 ± 2 °C for 30 min, and the formation of *p*-nitrophenol was determined spectrophotometrically at 410 nm. One unit of lipase activity was defined as the amount of enzyme that liberates 1 µmoL of p-nitrophenol per minute under the assay conditions.

#### Protease activity

Protease activity was determined according to Cupp-Enyard^29^. Briefly, 25 µL of ECE was mixed with 130 µL of substrate solution (0.65% casein in 50 mM Tris-HCl, pH 7.5) and incubated at 37 ± 2 °C for 10 min. Subsequently, 130 µL of 110 mM trichloroacetic acid was added, and the mixture was incubated for an additional 20 min at 37 °C, followed by centrifugation at 12,000 rpm for 10 min. From the supernatant, 50 µL was mixed with 125 µL of 500 mM sodium carbonate and 25 µL of Folin-Ciocalteu’s reagent. The reaction mixture was incubated at 37 °C for 30 min, and absorbance was measured at 660 nm. The tyrosine concentration was then estimated, and one unit of protease activity was defined as the amount of enzyme that releases 1 µmoL of tyrosine per minute under the assay conditions.

#### Chitinase activity (N-Acetylglucosaminidase)

Chitinase activity was measured in the ECE by following the procedure previously described by Mejía et al.^14^. Briefly, 20 µL of ECE was mixed with 100 µL of *p*-nitrophenyl-N-acetyl-β-D-glucosaminide (*p*NP-GlcNAc) solution (1 mg/mL in 0.1 M citrate buffer, pH 5.5). The reaction mixture was incubated at 37 ± 2 °C for 30 min and stopped with 150 µL of NaOH-glycine buffer (pH 8.5). Absorbance was measured at 400 nm, and the concentration of p-nitrophenol was estimated. One unit of chitinase activity was defined as the amount of enzyme that releases 1 µmoL of p-nitrophenol per minute under the assay conditions.

#### Glutathione peroxidase activity

Glutathione peroxidase (POX) activity was performed according to Débora et al.^30^. Briefly, 300 µL of crude enzyme preparation was added to a solution composed of 50 mm phosphate buffer (pH 6.5), 0.38 mM EDTA, 0.95 mM azide (to inhibit catalase), 1 mM glutathione, 0.12 mM nicotinamide adenine dinucleotide phosphate (NADPH), 3.2 U glutathione reductase, 0.02 mM DL-dithiothreitol, and 0.0007% hydrogen peroxide. The decrease in absorbance was monitored for 5 min at 340 nm in a spectrophotometer. Glutathione peroxidase activity was expressed as U/gds, where one unit corresponds to the amount of enzyme that catalyzes the oxidation of 1 µmoL of reduced glutathione to oxidized glutathione per minute at 25 °C and pH 7.0.

#### Superoxide dismutase activity

Superoxide dismutase (SOD) activity was determined using the method described by Nunes et al.^31^. Briefly, 50 µL of enzymatic extract was added to a reaction mixture containing 13 mM L-methionine, 75 µM nitro blue tetrazolium (NBT), 100 µM EDTA, and 2 µM riboflavin in 50 mm potassium phosphate buffer (pH 7.8). The reaction occurred in a chamber illuminated with a 30 W fluorescent lamp at 25 °C. The reaction was initiated by switching on the light and stopped after 5 min by turning it off. The formation of blue formazan from NBT photoreduction was measured as an increase in absorbance at 560 nm. One unit of SOD activity was defined as the amount of enzyme required to inhibit 50% of NBT photoreduction compared to control tubes without the extract and is expressed as unit of enzyme activity per gram of dry substrate (U/gds).

### Determination of phytochemicals

Phytochemical analysis of the extracts was evaluated individually. Total polyphenol content was determined based on the method of Medoua et al.^32^, with absorbance measured at 725 nm and results expressed as mg of gallic acid equivalents (GAE). Total flavonoids were quantified by the aluminum chloride colorimetric technique^33^, with absorbance measured at 415 nm and represented as mg of quercetin equivalents (QE)/g of dry extracts. The total alkaloid content was measured spectrophotometrically^34^, based on the reaction with bromocresol green, with absorbance recorded at 420 nm. Oxymatrine was used for the calibration curve, and total alkaloid content is reported as mg oxymatrine equivalent per g dry substrate (mg/gds).

### Analysis of total fatty acids (TFAs)

After solid-state fermentation of agro-industrial residues for screening (taro pomace) and optimization studies (orange pomace, banana pomace, sunflower cake, tomato pomace, taro pomace, or molokhia stalk), fungal biomass was separated from the substrate by washing with buffer, followed by solvent extraction with a chloroform-methanol mixture (2:1 v/v). Lipid extraction was performed according to the Folch technique^35^. Briefly, 1 g of fungal biomass was homogenized and extracted with 20 mL of chloroform-methanol (2:1 v/v), vigorously mixed to solubilize lipids, and allowed to undergo initial phase separation. Subsequently, 4 mL of deionized water was added to facilitate phase separation. The lower chloroform phase, containing the lipids, was collected, evaporated under reduced pressure, and used to determine total fatty acids.

###  Taxonomic studies and molecular identification of the selected fungal strain MORSY-27

The morphological characteristics of the selected isolate were evaluated following standard mycological procedures. These included assessments of colony texture, sporulation intensity, conidial color, and the abundance, texture, and color of mycelia. The presence and color of soluble pigments and exudates, the colony reverse color, and the overall growth rate were also examined across different culture media. Additional evaluations considered temperature and pH requirements, as well as microscopic features^36–39^. Enzyme activities were assessed using the API ZYM test kit (bioMérieux SA, 69280 Marcy-l’Etoile, France). For fungal characterization, the isolate was centrally inoculated onto several standard growth media, including potato dextrose agar (PDA), Sabouraud dextrose agar (SDA), yeast extract sucrose agar (YES), malt extract agar (MEA), peptone yeast extract agar (PYA), and Czapek yeast autolysate agar (CYA). Colour characteristics were determined using the Methuen Handbook of Colour (3rd edition) and the Colour Harmony Manual (4th edition)^40,41^. Scanning electron microscopy (SEM) was performed according to protocols routinely used in our laboratory^27,42,43^.

Fungal strain MORSY-27 was cultivated on PDA medium for 5 days at 28 °C and identified using a molecular approach involving DNA amplification and sequencing of the internal transcribed spacer (ITS) region. Genomic DNA was extracted from the pure culture using the DNeasy Plant Mini Kit (QIAGEN, USA) according to the manufacturer’s protocol. The extracted DNA was amplified by polymerase chain reaction (PCR) using the HotStarTaq Master Mix Kit (QIAGEN, USA) with ITS1 and ITS4 primers. The resulting PCR products were purified and submitted to a commercial sequencing service. Sequence data were edited with Lasergene Software SeqMan (DNAStar Inc.). Next, relatives were identified by comparing rRNA genes in the National Center for Biotechnology Information (NCBI) GenBank database using the Basic Local Alignment Search Tool (BLAST; http://www.ncbi.nlm.nih.gov). This comparison was performed using MEGA11 and ClustalW to generate a matrix, and the resulting sequences were subsequently deposited in NCBI GenBank^44–48^. Phylogenetic analysis was performed using the neighbor-joining method in MEGA11 software with 1,000 bootstrap replicates^49–51^.

### Impacts of agro-industrial residues, temperature, and pH on bioactive metabolite production by *Geomyces* sp. MORSY-27

 Different agro-industrial residues, including orange pomace (OP), taro pomace (TP), banana pomace (BC), sunflower cake (SFC), tomato pomace (ToP), and molokhia stalk (MS), were evaluated as production media for phytochemicals (phenolics, terpenoids, flavonoids, and alkaloids), cuticle-degrading enzymes (extracellular lipases, proteases, and chitinases), antioxidant enzymes (superoxide dismutase and glutathione peroxidase), and total fatty acids (TFAs) content without any supplements. These natural culture media were also evaluated for their pesticidal activity against *A. ipsilon* and *T. urticae*. Briefly, 10 g of each agro-industrial residue was sieved to 0.5 mm and put individually into two groups of 250 mL Erlenmeyer flasks. In the first group, substrates were moistened to an initial moisture content (IMC) of 55%, inoculated with a spore suspension of *Geomyces* sp. MORSY-27 at 2 × 10^7^ spores/gds, and incubated at 10, 20, 25, and 30 °C for 10 days to determine the optimal temperature. In the second group, substrates were adjusted to different IMCs (50, 55, 60, and 70%) and inoculated as described above, then incubated at the optimal growth temperature (20 °C) for 10 days. After incubation, bioactive metabolites were extracted and analyzed as previously described.

### Evaluation of the pesticidal activity of *Geomyces* sp. MORSY-27 metabolites produced on different agro-industrial residues

#### Insecticidal activity

Larvae of *A. ipsilon* were obtained from organic greenhouses at the Faculty of Agriculture, Cairo University, Egypt. The insect culture was maintained for more than 10 generations on castor-oil plant leaves (*Ricinus communis* L., Malpighiales: Euphorbiaceae) under controlled laboratory conditions (22 ± 2 °C, 65 ± 5% relative humidity, and a 16 L:8D photoperiod). Castor leaves were thoroughly washed, air-dried, and replaced daily. Rearing was conducted without exposure to insecticides or chemical contaminants. Initially, ten larvae were placed in each glass jar (10 × 9 × 18 cm). To avoid cannibalism, fourth-instar larvae were transferred individually to 120 mL plastic cups. Fully developed larvae were monitored daily until pupation, after which prepupae were transferred to separate jars containing moist sawdust. A black muslin cloth was used as the oviposition surface. Newly emerged adults were placed in oviposition jars at a 1:2 male-to-female ratio, and cotton tufts soaked in a 10% honey solution were provided as a food source for adults^52^.

The toxicity of *Geomyces* sp. MORSY-27 metabolites, produced on different agro-industrial residues (OP, TP, BP, SFC, ToP, and MS), was evaluated against second-instar larvae of *A. ipsilon*. Each crude extract was dissolved in 10% dimethyl sulfoxide (DMSO) to prepare fresh stock solutions at the desired concentrations. The castor leaf-dipping method was used, in which leaves were immersed in each treatment solution for 20 s at concentrations of 1.0, 2.0, and 4.0%, as determined from preliminary trials. Each concentration was tested in 25 replicates, with a single newly molted second-instar larva placed in a separate 6-cm-diameter Petri dish for each replicate. A control group was maintained, fed untreated leaves treated with 10% DMSO. Treated leaves were provided for feeding over three days, after which larvae were supplied daily with fresh, untreated castor leaves. All experiments were conducted under controlled laboratory conditions (22 ± 2 °C, 65 ± 5% relative humidity, 16 L:8D photoperiod). Larvae were monitored daily to record any deformities, the duration of larval and pupal stages, larval and pupal mortality, and adult emergence.

#### Acaricidal activity

*Tetranychus urticae* was reared in the laboratory for multiple generations on green copper leaves of *Acalypha wilkesiana* Müll.Arg. (Malpighiales: Euphorbiaceae), originally collected from infested castor bean leaves (*Ricinus communis* L., Malpighiales: Euphorbiaceae). For rearing, individual *A. wilkesiana* leaves were arranged singly, upside down, on moist cotton wool in 9-cm-diameter Petri dishes. To sustain the population, infested leaves were consistently transferred onto fresh *A. wilkesiana* leaves. Both culture and experimental conditions were maintained at 26 ± 2 °C and 65 ± 5% relative humidity.

For the egg toxicity assay, ten 5-day-old female mites were placed on 3-cm-diameter *A. wilkesiana* leaf discs positioned upside-down on moistened cotton wool in Petri dishes. The females were left for 24 h to deposit eggs; after which they were removed, and the deposited eggs were then sprayed using a glass atomizer directly with selected concentrations (0.5, 1.0, and 2.0%) of the tested extracts, prepared as described above. Concentrations that caused 10–99% mortality in preliminary tests were selected for the experiment. Each treatment was replicated five times, with 50 eggs per replicate. Eggs were incubated for approximately six days until hatching, and the number of unhatched eggs was recorded. The same procedure was applied to assess the toxic effects of the tested extracts on adult females. In this case, each concentration included 20 females per replicate, and mortality was recorded at 24 and 96 h post-treatment. Eggs deposited by treated females were collected daily for four consecutive days, and hatchability was monitored for each day as well as cumulatively over the four-day oviposition period. Each bioassay was conducted twice on separate dates. This methodology followed^53^, and control groups were maintained under the same conditions and received no treatment, only a 10% DMSO solution.

### GC-MS analysis

*Geomyces* sp. MORSY-27 was selected as the optimal fungal strain, and sunflower cake (SFC) was chosen as the most suitable substrate based on its suitability for supporting fungal growth and targeted metabolite production. The culture was maintained at an initial moisture content of 55% and an incubation temperature of 20 °C. Under these optimal conditions, the fungal culture was extracted with ethanol as mentioned above. For GC-MS analysis, fungal metabolites were extracted in ethanol for three hours with shaking at 120 rpm at room temperature. The extract was then concentrated by evaporating the solvent under reduced pressure using a rotary evaporator until dry. The dried extract was dissolved in chloroform (CHCl3) and then analyzed by GC-MS. GC-MS analysis was performed using a Thermo Scientific Trace GC Ultra/ISQ Single Quadrupole MS equipped with a TG-5MS fused silica capillary column (30 m × 0.25 mm, 0.1 μm film thickness). An electron ionization system with an ionization energy of 70 eV was used, and helium served as the carrier gas at a constant flow rate of 1mL/minute. The MS transfer line and injector temperatures were set at 280 °C. The oven temperature was programmed as follows: an initial temperature of 50 °C (held for 2 min), ramped to 150 °C at 7 °C/minute, then to 270 °C at 5 °C/minute (held for 2 min), and finally to 310 °C at 3.5 °C/minute (held for 10 min). Quantification of the identified components was investigated using the percentage relative peak area, while tentative compound identification was achieved by comparing relative retention times and mass spectra with those in the NIST and Wiley library data of the GC-MS system.

### Statistical analysis

All in vitro experimental data are expressed as mean ± standard error (SE) from three independent replicates. Mortality and egg hatchability percentages of *T. urticae* were corrected using Abbott’s formula^54^. Statistical analyses for pesticidal bioassay data were performed using one-way ANOVA followed by Duncan’s multiple range test in SPSS software (version 26). Differences were considered statistically significant at *p* ≤ 0.05. Each bioassay was conducted twice to ensure reproducibility.

### Ethics approval and consent to participate

This study did not involve human or vertebrate animal experiments. The use of *A. ipsilon* and *T. urticae* was performed in accordance with relevant institutional and national guidelines and regulations.

## Results

### Isolation of endophytic fungi of wheat collected in different areas

In the current work, a total of 38 endophytic fungal isolates were obtained from wheat plants (Table S1). Fifteen isolates, designated MORSY-1 to MORSY-15, were isolated from Abu Ḥammad, Sharqia Governorate, located in the Nile Delta. Eleven isolates, coded MORSY-16 to MORSY-26, were recovered from Matai, El-Minia Governorate of Middle Egypt. The remaining twelve isolates, named MORSY-27 to MORSY-38, were obtained from Girga, Sohag Governorate of Upper Egypt (Fig. S1a, b, c, d; Table S1).

### Screening of endophytic fungi for the production of pesticidal metabolites using Taro pomace under solid-state fermentation

Taro pomace (TP), a nutrient-rich agro-industrial byproduct, provides an excellent solid substrate that enhances microbial growth and stimulates the biosynthesis of diverse secondary metabolites during solid-state fermentation. Its high content of carbohydrates, fibers, and micronutrients makes TP particularly suitable for supporting metabolite pathways associated with pesticidal activity. The ability of the isolated endophytic fungi to produce various pesticidal metabolites was assessed under solid-state fermentation (SSF) using taro pomace (TP) as the substrate. Since these substances are known to contribute to pesticidal activity, the initial screening of endophytic fungal isolates focused on the total synthesis of bioactive metabolites, including alkaloids, phenolics, flavonoids, terpenoids, fatty acids, and cuticle-degrading enzymes. To validate actual biocontrol activity, an isolate exhibiting substantial metabolite outcome was further evaluated in confirmatory insecticidal and acaricidal bioassays. The results of this screening are summarized in Table S1.

#### Phytochemicals and total fatty acids productivity

As shown in Table S1, the production of bioactive metabolites is considerably higher among the endophytic fungal isolates collected from the different regions of Egypt. The highest alkaloid yields were obtained from isolates MORSY-27 (61.15 ± 4.75 mg/gds) and MORSY-36 (59.20 ± 4.70 mg/gds) originating from Upper Egypt. These were followed by MORSY-26 (57.16 ± 4.52 mg/gds) from Middle Egypt and MORSY-10 (54.74 ± 4.10 mg/gds) from the Delta region. All isolates produced detectable levels of phenolic compounds, with the highest production observed in MORSY-27 (245.42 ± 8.90 mg/gds) from Upper Egypt, MORSY-21 (241.50 ± 8.16 mg/gds) from Middle Egypt, and MORSY-11 (240.17 ± 7.18 mg/gds) from the Delta region. The highest flavonoid content was detected in isolate MORSY-27 (92.84 ± 7.17 mg/gds), followed by MORSY-7, MORSY-23, and MORSY-31, which produced comparable levels (90.50–90.13 mg/gds). In terms of terpenoid production, MORSY-27 exhibited the greatest yield (69.18 ± 5.73 mg/gds), followed by MORSY-15 (60.13 ± 4.69 mg/gds) and MORSY-1 (56.47 ± 4.57 mg/gds). Similarly, total fatty acid production was highest in MORSY-27 (57.39 ± 4.34 mg/gds), with substantial amounts also recorded in MORSY-26 (52.37 ± 4.30 mg/gds), MORSY-3 (50.50 ± 4.50 mg/gds), and MORSY-37 (50.19 ± 4.20 mg/gds). The isolate MORSY-27 from Upper Egypt showed the greatest and most consistent productivity across all categories of bioactive metabolites.

#### Cuticle-degrading enzymes productivity

As shown in Table S1, the endophytic fungal isolates exhibited varying capacities to produce extracellular hydrolytic enzymes, which are associated with biocontrol potential. The highest chitinase activities, which play a key role in degrading insect cuticles and fungal cell walls, were recorded in isolates from Upper Egypt, particularly MORSY-27 (847.65 ± 19.30 U/gds) and MORSY-36 (824.76 ± 19.47 U/gds). High chitinase yield was also observed in MORSY-17 (729.18 ± 16.00 U/gds) from Middle Egypt and MORSY-1 (653.14 ± 14.25 U/gds) from the Delta region (Table S1). Regarding protease production, which further contributes to cuticle degradation and pathogenicity, the highest activities were detected in MORSY-27 (1020.49 ± 17.45 U/gds), MORSY-18 (1017.65 ± 16.85 U/gds), and MORSY-3 (1010.48 ± 17.33 U/gds). Several additional isolates, including MORSY-6, MORSY-9, and MORSY-28, also exhibited strong proteolytic activity (Table S1). For lipase production, the highest activities were recorded in MORSY-27 (280.31 ± 7.85 U/gds) from Upper Egypt, followed by MORSY-3 (279.20 ± 8.30 U/gds) from the Delta region and MORSY-17 (270.15 ± 7.93 U/gds) from Middle Egypt (Table S1). Notably, MORSY-27 consistently showed the highest production of all three extracellular enzymes: chitinase, protease, and lipase, highlighting its strong potential as a promising biocontrol agent.

### Phenotypic, chemotypic, and molecular identification of the selected endophytic isolate MORSY-27

The endophytic fungal isolate MORSY-27 was identified using a combination of morphological, physiological, biochemical, and molecular analyses. Its cultural characteristics on different media exhibited distinct colony morphologies (Table S2). On potato dextrose agar, colonies were soft, fluffy, and white with a light green center that gradually turned bright yellow. On Sabouraud dextrose agar, colonies appeared velvety and light brown, with a yellow-brown reverse. Yeast sucrose agar supported abundant sporulation, producing granular, pastel-brown colonies, whereas malt extract agar yielded flat, powdery colonies with dull olive pigmentation. Growth on peptone yeast extract agar yielded wrinkled, greyish-olive colonies. On Czapek yeast extract agar, the fungus formed circular, powdery colonies with a dark green reverse and released a diffusible brown pigment. Physiologically, the strain was capable of growing between 4 °C and 34 °C (optimum 10–25 °C) and at pH levels of 4.0–8.0 (optimum 5.0–5.5.0.5), with no growth observed at 37 °C.

**Fig. 1:**
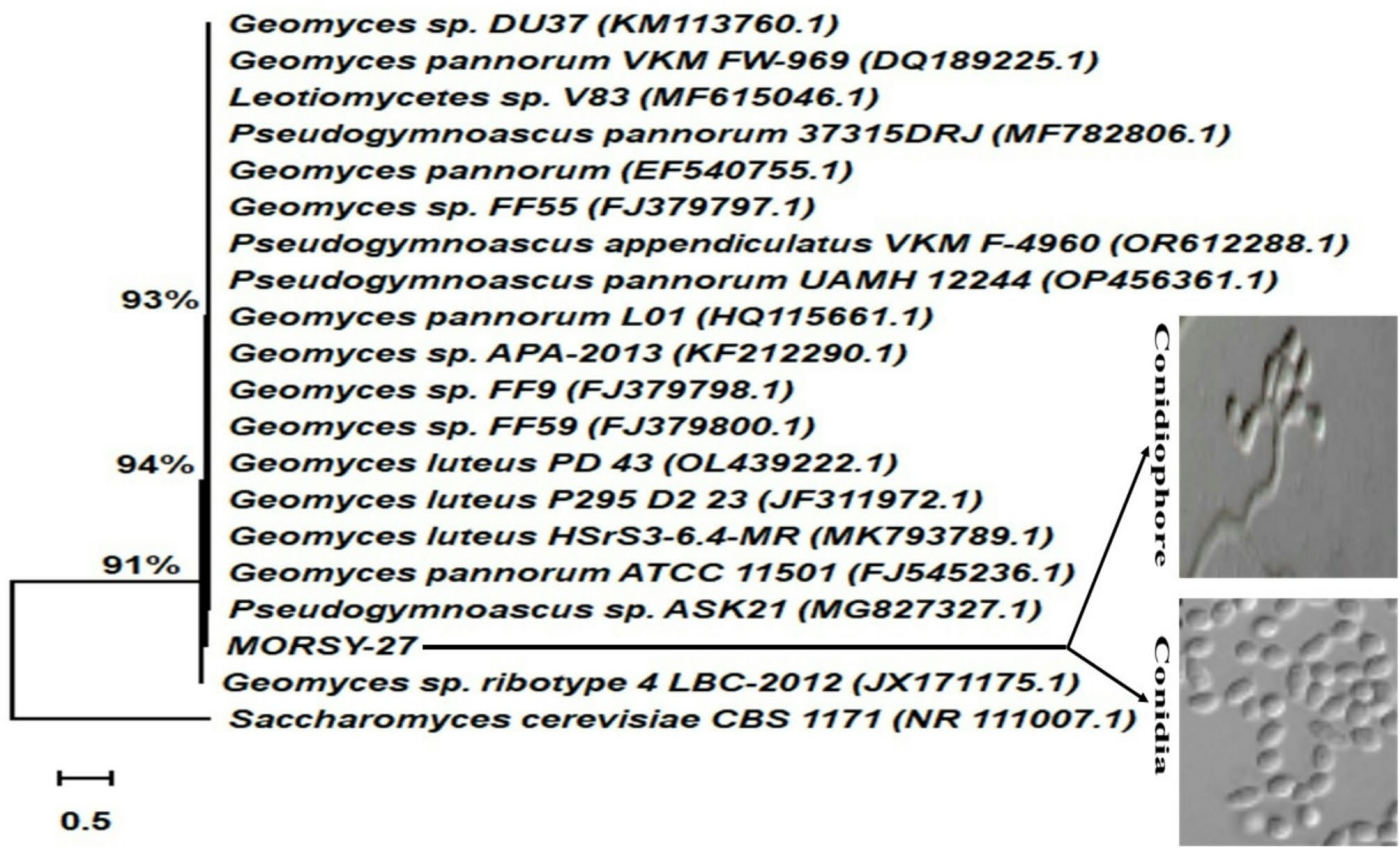
Microscopic characteristics and phylogenetic tree of the selected fungal strain MORSY**-**27 generated by the neighbor-joining method based on rDNA-ITS sequences analysis. *Saccharomyces cerevisiae* was used as an outgroup.

Microscopic examination revealed hyaline, erect, smooth-walled conidiophores (16–20 × 4.5–5.8 μm) arranged in verticils of 3–4 branches, forming characteristic tree-like sporulating structures. The conidia were aseptate, smooth, and pyriform to clavate in shape (3.5–8.2 × 2.7–5.2 μm), produced in short chains at the tips of the conidiophores (Fig. 1). Biochemically, isolate MORSY-27 displayed strong enzymatic activity, producing phosphatase, β-glucosidase, esterase, lipase, N-acetyl-β-glucosaminidase, proteinase, α-mannosidase, and α-fucosidase. However, it lacked valine, cystine, and leucine arylamidase, as well as α-chymotrypsin activity (Table S2). The morphological traits, particularly its simple conidiophores and psychrophilic nature, were consistent with a member of the genus *Geomyces*.

Molecular identification of strain MORSY-27 using ITS1/ITS4 primers generated a 534 bp rDNA-ITS fragment, which was subsequently sequenced and deposited in GenBank under the assigned accession number PX588620. The obtained sequence was analyzed using the BLAST algorithm and compared against entries in the NCBI Nucleotide Sequence Database. BLAST analysis performed through the *blastn* tool (GenBank, NCBI) and the corresponding phylogenetic assessment (Fig. 1) indicated that strain MORSY-27 belonged to the phylum *Ascomycota*, class *Leotiomycetes*, order *Helotiales*, family Myxotrichaceae, and genus *Geomyces*. As shown in Fig. 1, isolate MORSY-27 clustered with *Geomyces* sp. FF9 (99.44% similarity), as well as *Geomyces* sp. APA-2013, *Geomyces* sp. FF55, *Geomyces pannorum* L01, *Pseudogymnoascus appendiculatus* VKM F-4960, *Pseudogymnoascus pannorum* UAMH 12,244, and *Leotiomycetes* sp. V83 (99.25% similarity). The selected strain MORSY-27 was initially identified based on its morphological, physiological, and biochemical properties, followed by phylogenetic analysis of its rDNA ITS region. Based on these combined criteria, isolate MORSY-27 is identified and designated as *Geomyces* sp. MORSY-27.

### Evaluation of agro-industrial residues, initial moisture content, and incubation temperature on the production of pesticidal metabolites

The selection of diverse agro-industrial residues as fermentation media was driven by their compositional variability and their potential to stimulate different biosynthetic pathways in endophytic fungi. Each residue offers a unique profile of carbohydrates, fibers, proteins, lipids, and micronutrients, which can influence fungal physiology and the spectrum of metabolites produced. For instance, fruit pomaces such as orange pomace (OP), taro pomace (TP), and banana pomace (BP) are rich in simple sugars and pectin that support rapid fungal colonization and secondary metabolite induction. Oilseed residues, such as sunflower cake (SFC), have high lipid and protein content, which favors the secretion of cuticle-degrading enzymes and the production of fatty acids. Similarly, tomato pomace (ToP) and molokhia stalk (MS) provide substantial amounts of cellulose, hemicellulose, and bioactive precursors that can modulate pathways involved in the biosynthesis of alkaloids, phenolics, and terpenoids.

In selecting these substrates, the rationale was to cover a broad nutritional spectrum that could differentially enhance phytochemical production, extracellular enzymatic activity, and total fatty acid yields under solid-state fermentation (SSF). The concentrations used and the optimization parameters, including incubation temperature, initial moisture content, substrate-to-inoculum ratio, and fermentation period, were derived from prior SSF fermentation studies with filamentous fungi. Previous investigations have consistently shown that moderate temperatures (20–25 °C) maximize fungal enzymatic activity while preventing thermal stress and that maintaining substrate moisture between 50 and 70% ensures adequate water availability without compromising aeration. These ranges also promote efficient diffusion of metabolites within the solid matrix. Moreover, the selected substrate quantities and fungal inoculum densities were chosen based on preliminary trials demonstrating stable growth kinetics and reproducible biochemical outputs. Therefore, the adopted conditions represent evidence-based parameters aligned with documented SSF performance for bioactive metabolite generation. Different agro-industrial residues, including OP, TP, BC, SFC, ToP, and MS, were evaluated as production media for phytochemicals, cuticle-degrading enzymes, antioxidant enzymes, and total fatty acid (TFA) content without supplementation.

#### Phytochemical components productivity

As shown in Table 1, *Geomyces* sp. MORSY-27 produced considerable quantities of key phytochemicals, including phenolics, terpenoids, flavonoids, and alkaloids, as well as total fatty acids, when cultivated on six agro-industrial residues (OP, TP, BP, SFC, ToP, and MS). The highest yields of all metabolites were achieved under optimized conditions of 20 °C and an initial moisture content (IMC) of 55%. Under these optimal parameters, SFC and TP substrates proved particularly effective, yielding the highest levels of phenolics (282.74 ± 6.71 and 270.88 ± 6.83 mg/gds, respectively), terpenoids (86.12 ± 5.29 and 75.18 ± 5.20 mg/gds), flavonoids (119.61 ± 7.45 and 105.73 ± 6.83 mg/gds), and alkaloids (70.82 ± 4.83 and 60.11 ± 4.15 mg/gds). Any deviation from these optimal parameters, either lower (10 °C, 50% IMC) or higher (25–30 °C, 70% IMC), resulted in marked reductions in phytochemical production across all tested substrates.

**Table 1:** Productivity of phytochemicals and total fatty acids by *Geomyces* sp. MORSY27 on different agro-industrial residues under various initial moisture contents and temperature.

Cultural conditions (mg/gds)								Aqueous extract of fermented
Initial moisture content (IMC, %) at 20 °C				Temperature (°C) at 55% IMC				
70	60	55	50	30	25	20	10	
Phenolic content (mg/gds)								
82.41 ± 3.42 168.10 ± 5.37 113.89 ± 3.96 174.22 ± 5.35 126.28 ± 4.22 111.73 ± 3.74	128.30 ± 4.37 209.16 ± 5.94 150.34 ± 4.71 218.00 ± 5.93 173.57 ± 5.31 159.60 ± 4.80	169.50 ± 5.10 270.88 ± 6.83 182.67 ± 5.38 282.74 ± 6.71 211.78 ± 5.84 195.23 ± 5.72	132.70 ± 4.11 224.64 ± 5.97 169.33 ± 0.25 241.25 ± 6.43 196.83 ± 5.65 180.22 ± 5.12	72.50 ± 3.00 160.46 ± 5.20 85.11 ± 3.42 176.20 ± 5.54 118.32 ± 4.42 100.45 ± 4.20	116.24 ± 4.46 254.42 ± 6.42 136.96 ± 4.85 265.80 ± 6.47 169.47 ± 5.59 163.50 ± 5.00	169.50 ± 5.10 270.88 ± 6.83 182.67 ± 5.38 282.74 ± 6.71 211.78 ± 5.84 195.23 ± 5.72	115.76 ± 4.30 198.50 ± 5.87 137.46 ± 4.78 218.14 ± 6.24 160.75 ± 5.41 148.13 ± 5.23	Orange pomace (OP) Taro pomace (TP) Banana pomace (BP) Sunflower cake (SCF) Tomato pomace (ToP) Molokhia stalk (MS)
Terpenoid (mg/gds)								
20.08 ± 1.37 41.12 ± 2.69 25.38 ± 1.46 50.60 ± 3.63 37.55 ± 2.29 32.14 ± 1.68	30.78 ± 2.04 60.55 ± 4.23 34.27 ± 2.42 71.10 ± 4.78 49.13 ± 3.25 40.81 ± 2.66	40.51 ± 2.86 75.18 ± 5.20 44.17 ± 3.19 86.12 ± 5.29 60.10 ± 4.16 52.94 ± 3.77	35.70 ± 2.43 62.01 ± 4.10 39.85 ± 2.54 74.23 ± 5.13 52.21 ± 3.86 44.70 ± 3.20	23.50 ± 1.62 52.62 ± 3.83 29.35 ± 1.90 52.70 ± 3.84 40.21 ± 2.50 35.18 ± 2.41	34.50 ± 2.25 75.42 ± 4.65 38.67 ± 2.41 80.62 ± 5.63 56.40 ± 4.00 47.55 ± 3.34	40.51 ± 2.86 75.18 ± 5.20 44.17 ± 3.19 86.12 ± 5.29 60.10 ± 4.16 52.94 ± 3.77	30.25 ± 2.05 64.29 ± 4.28 36.42 ± 2.22 70.00 ± 4.75 45.28 ± 3.00 39.50 ± 2.34	Orange pomace (OP) Taro pomace (TP) Banana pomace (BP) Sunflower cake (SCF) Tomato pomace (ToP) Molokhia stalk (MS)
Flavonoid (mg/gds)								
20.54 ± 1.34 40.18 ± 3.11 25.17 ± 1.78 59.30 ± 4.20 32.62 ± 2.38 25.94 ± 1.43	30.18 ± 2.10 57.32 ± 4.15 33.16 ± 2.30 62.24 ± 5.12 44.57 ± 3.36 39.85 ± 2.50	63.05 ± 4.52 105.73 ± 6.83 67.98 ± 5.19 119.61 ± 7.45 83.70 ± 6.45 74.89 ± 5.30	37.13 ± 3.12 65.58 ± 5.27 40.21 ± 3.23 78.36 ± 6.06 52.46 ± 3.72 46.78 ± 0.34	20.00 ± 1.59 35.28 ± 2.14 23.25 ± 1.65 47.80 ± 3.82 26.72 ± 179 24.95 ± 1.71	40.39 ± 3.24 63.50 ± 5.18 42.71 ± 3.43 70.24 ± 5.29 50.21 ± 3.66 46.83 ± 3.52	63.05 ± 4.52 105.73 ± 6.83 67.98 ± 5.19 119.61 ± 7.45 83.70 ± 6.45 74.89 ± 5.30	55.00 ± 4.00 80.32 ± 6.00 59.58 ± 4.21 84.60 ± 6.43 75.91 ± 5.26 60.67 ± 4.39	Orange pomace (OP) Taro pomace (TP) Banana pomace (BP) Sunflower cake (SFC) Tomato pomace (ToP) Molokhia stalk (MS)
Alkaloid (mg/gds)								
20.75 ± 1.39 32.13 ± 2.17 21.50 ± 1.34 40.27 ± 2.48 30.67 ± 2.00 26.91 ± 1.53	26.50 ± 1.52 46.28 ± 3.57 32.46 ± 2.18 50.08 ± 3.75 38.50 ± 2.46 37.68 ± 2. 39	39.28 ± 2.92 60.11 ± 4.15 45.89 ± 3.27 70.82 ± 4.83 56.17 ± 3.89 50.32 ± 3.56	31.62 ± 2.00 50.54 ± 3.63 41.92 ± 2.68 59.94 ± 4.15 43.76 ± 3.00 39.11 ± 2.49	19.77 ± 1.25 34.15 ± 2.40 25.48 ± 1.48 40.32 ± 2.51 29.61 ± 1.92 26.50 ± 1.54	30.93 ± 2.10 51.68 ± 3.71 37.75 ± 2.40 55.49 ± 3.86 44.70 ± 3.00 41.88 ± 2.54	39.28 ± 2.92 60.11 ± 4.15 45.89 ± 3.27 70.82 ± 4.83 56.17 ± 3.89 50.32 ± 3.56	30.72 ± 2.00 48.90 ± 3.20 36.51 ± 2.30 54.92 ± 3.91 43.80 ± 3.05 40.60 ± 2.83	Orange pomace (OP) Taro pomace (TP) Banana pomace (BP) Sunflower cake (SCF) Tomato pomace (ToP) Molokhia stalk (MS)
Total fatty acid (mg/gds)								
18.59 ± 1.14 40.28 ± 3.05 24.96 ± 1.43 40.50 ± 2.60 30.80 ± 2.23 25.96 ± 1.43	30.29 ± 2.30 50.65 ± 3.77 36.70 ± 2.55 54.02 ± 0.56 46.19 ± 3.40 37.80 ± 2.71	36.13 ± 2.52 65.58 ± 4.28 40.21 ± 2.70 78.36 ± 5.34 58.40 ± 3.97 46.78 ± 3.60	30.60 ± 2.24 57.99 ± 3.93 35.16 ± 2.50 60.20 ± 4.18 46.35 ± 3.25 38.80 ± 2.49	16.50 ± 0.98 30.24 ± 2.00 20.30 ± 1.17 40.62 ± 2.64 32.71 ± 2.05 24.19 ± 1.40	29.48 ± 1.72 57.94 ± 3.94 34.25 ± 2.18 63.18 ± 4.30 44.90 ± 3.15 38.50 ± 2.45	36.13 ± 2.52 65.58 ± 4.28 40.21 ± 2.70 78.36 ± 5.34 58.40 ± 3.97 46.78 ± 3.60	25.75 ± 1.47 48.21 ± 3.24 30.90 ± 2.19 59.83 ± 4.15 40.76 ± 2.50 35.40 ± 2.46	Orange pomace (OP) Taro pomace (TP) Banana pomace (BP) Sunflower cake (SCF) Tomato pomace (ToP) Molokhia stalk (MS)

#### Cuticle degrading and antioxidant enzymes

**Fig. 2:**
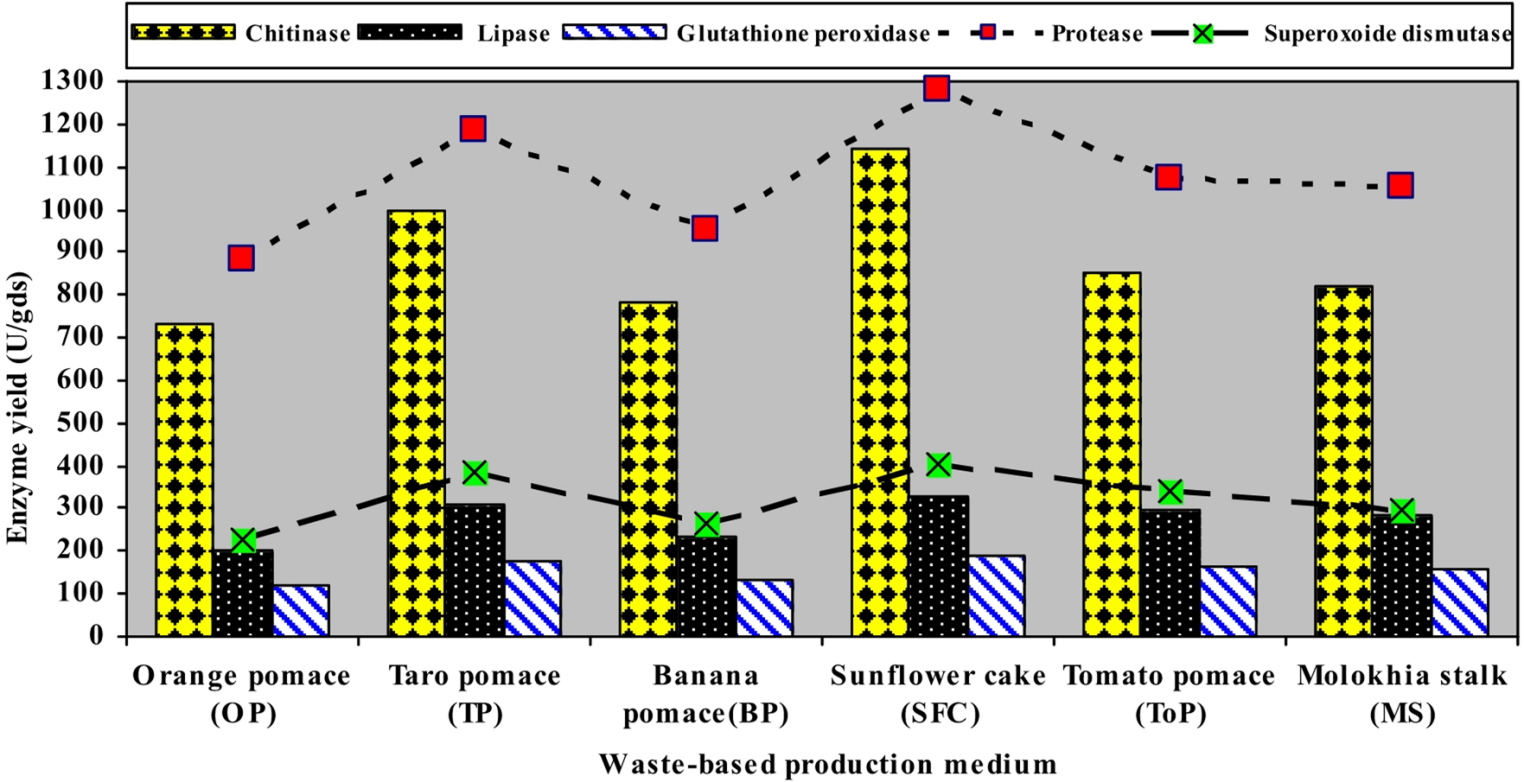
The effect of agro-industrial residues as low-cost production media on the yields of cuticle-degrading and antioxidant enzymes produced by *Geomyces* sp. MORSY-27.

As illustrated in Fig. 2, *Geomyces* sp. MORSY-27 exhibited strong production of cuticle-degrading and antioxidant enzymes across the agro-industrial residues tested. Sunflower cake (SFC) proved to be the most effective substrate, supporting the highest activities of lipase (326.38 U/gds), protease (1281.74 U/gds), and chitinase (1140.52 U/gds). These activities were substantially greater than those obtained from other residues, such as orange pomace, which yielded 200.56, 884.53, and 733.96 U/gds, respectively. Intermediate enzyme activities were observed on taro pomace, tomato pomace, banana peels, and molokhia stalk, reflecting clear substrate-dependent differences in enzyme synthesis. A similar trend was observed for antioxidant enzymes, whose activities increased with substrate type. The highest levels of superoxide dismutase (SOD) and glutathione peroxidase (GPx) were obtained in SFC medium (401.58 and 190.45 U/gds, respectively), followed by taro pomace (385.17 and 178.62 U/gds, respectively). Collectively, these findings highlight sunflower cake (SFC) as the most efficient and cost-effective substrate for enhancing both cuticle-degrading and antioxidant enzymes production by *Geomyces* sp. MORSY-27 under solid-state fermentation conditions.

#### Insecticidal activity of geomyces sp. MORSY-27

**Fig. 3:**
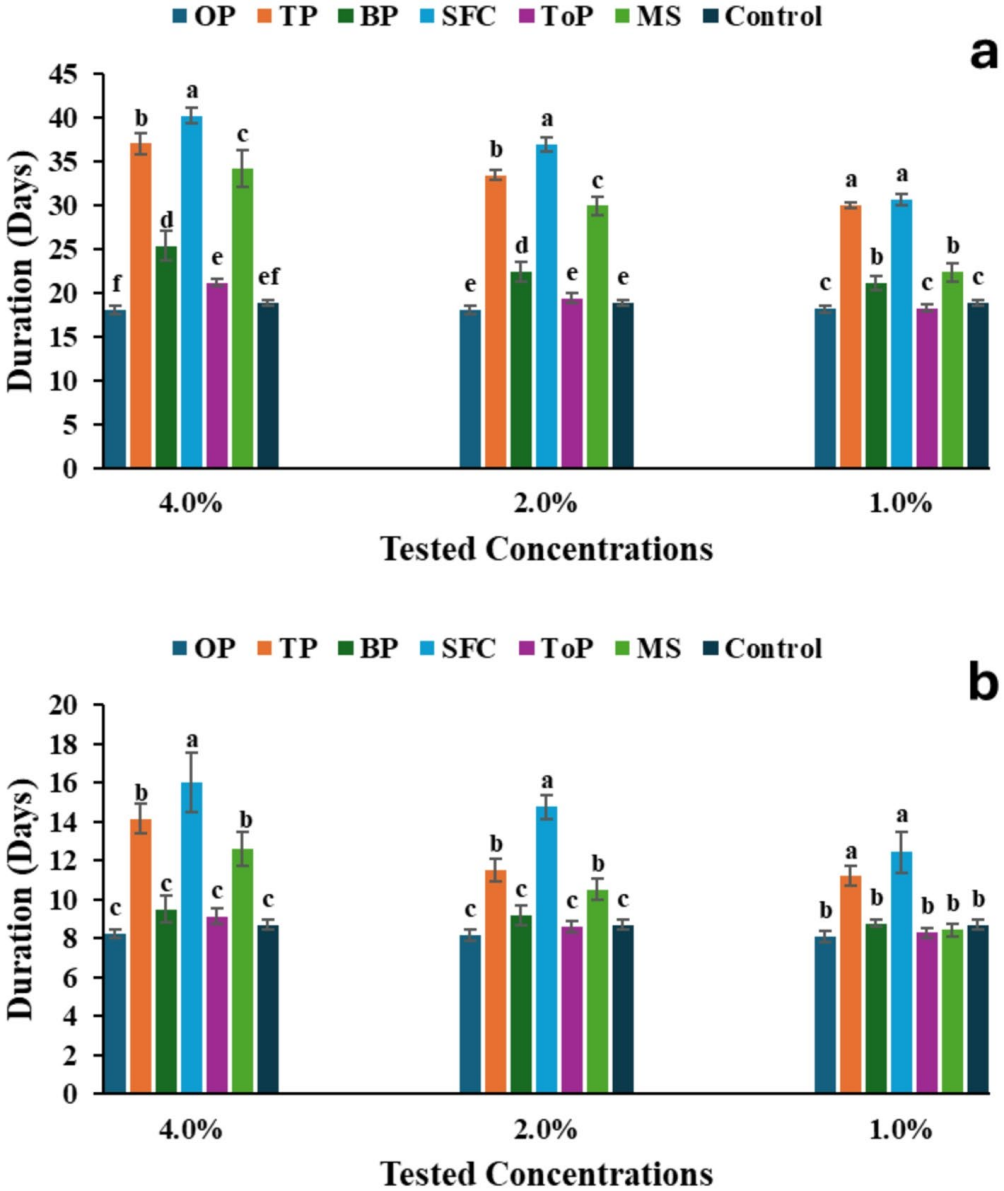
Effect of *Geomyces* sp. MORSY-27 extracts, produced on various agro-industrial residues [orange pomace (OP), taro pomace (TP), banana pomace (BP), sunflower cake (SFC), tomato pomace (ToP), and molokhia stalk (MS)], on **(a)** larval duration and **(b)** pupal duration of *A. ipsilon* at different concentrations. Mean values (± SE) with different letters within the same concentration are significantly different (*P* ≤ 0.05, ANOVA, Duncan’s test).

Extracts of *Geomyces* sp. MORSY-27 grown on different agro-industrial residues significantly influenced the larval duration of *A. ipsilon* (Fig. 3a). Across all tested concentrations (1.0, 2.0, and 4.0%), treated larvae exhibited prolonged development compared to the control. At 4.0%, the SFC extract produced the greatest extension in larval duration (41 days), followed by TP and MS, whereas the control group exhibited the shortest duration (18 days). A similar trend was observed at 2.0%, with the SFC extract again inducing the longest developmental period (38 days). At the lowest concentration (1.0%), ToP and BP resulted in the shortest larval durations (18–22 days), while TP and SFC extracts produced the longest (31–33 days). Overall, formulations from SFC and TP consistently caused the most significant developmental delay at all tested concentrations, and larval duration increased proportionately with the applied concentration (Fig. 3a).

Similarly, the pupal duration of *A. ipsilon* was significantly impacted by extracts of *Geomyces* sp. MORSY-27 at all tested concentrations (Fig. 3b). At the highest concentration, larvae treated with the SFC extract exhibited the longest pupal period, followed by those treated with TP and MS, whereas the control group showed the shortest duration. A comparable trend was observed at 2.0%, where SFC again produced the most pronounced extension in pupal duration (14 days), significantly exceeding all other treatments and the control. Although pupal duration decreased slightly at 1.0%, the SFC extract still resulted in the longest duration (12 days), while ToP extract and the control recorded the shortest periods (approximately 8–9 days).

**Fig. 4:**
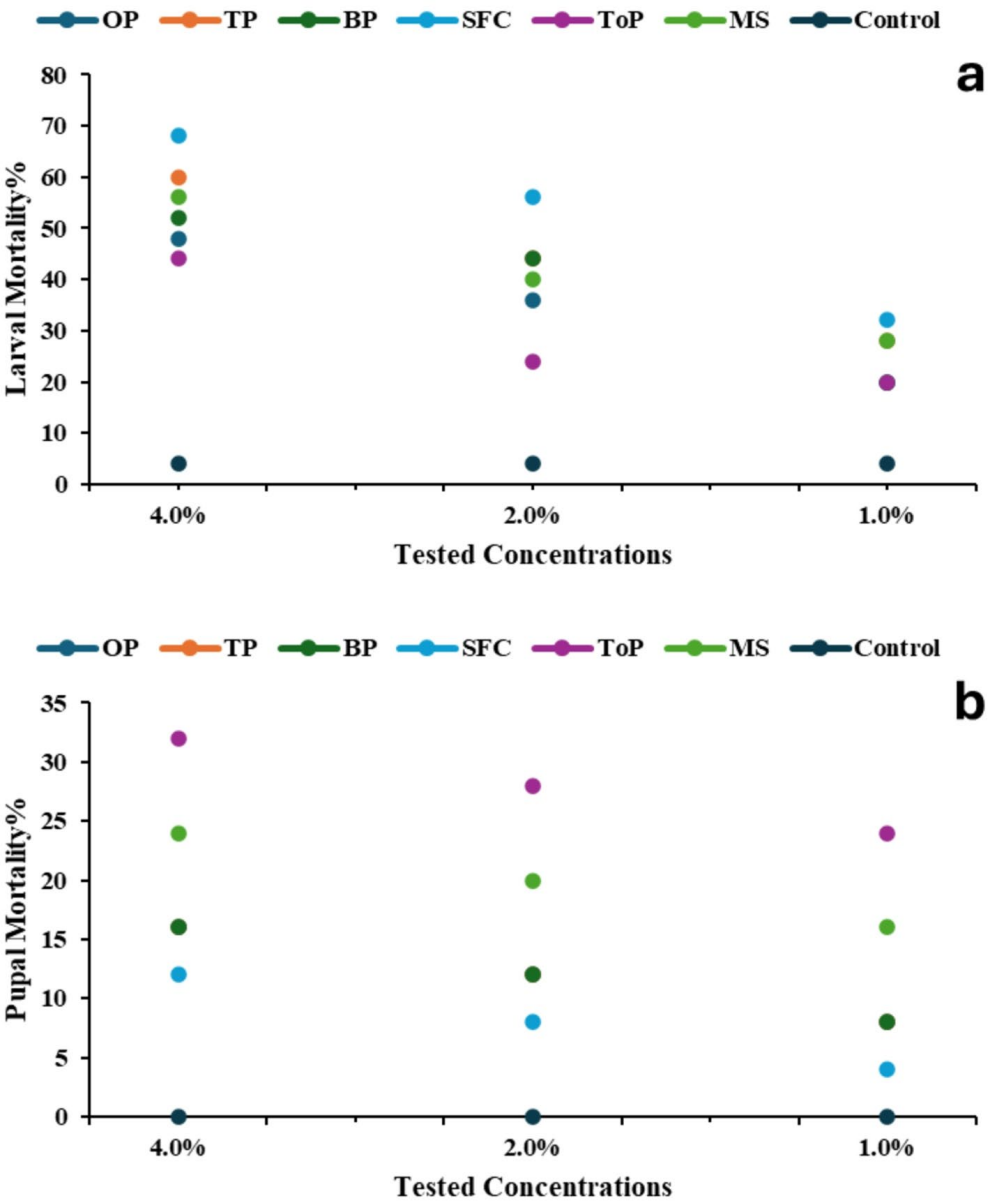
Effect of *Geomyces* sp. MORSY-27 extracts, produced on different agro-industrial residues [orange pomace (OP), taro pomace (TP), banana pomace (BP), sunflower cake (SFC), tomato pomace (ToP), and molokhia stalk (MS)], on **(a)** larval mortality and **(b)** pupal mortality of *A. ipsilon* at different concentrations.

**Fig. 5:**
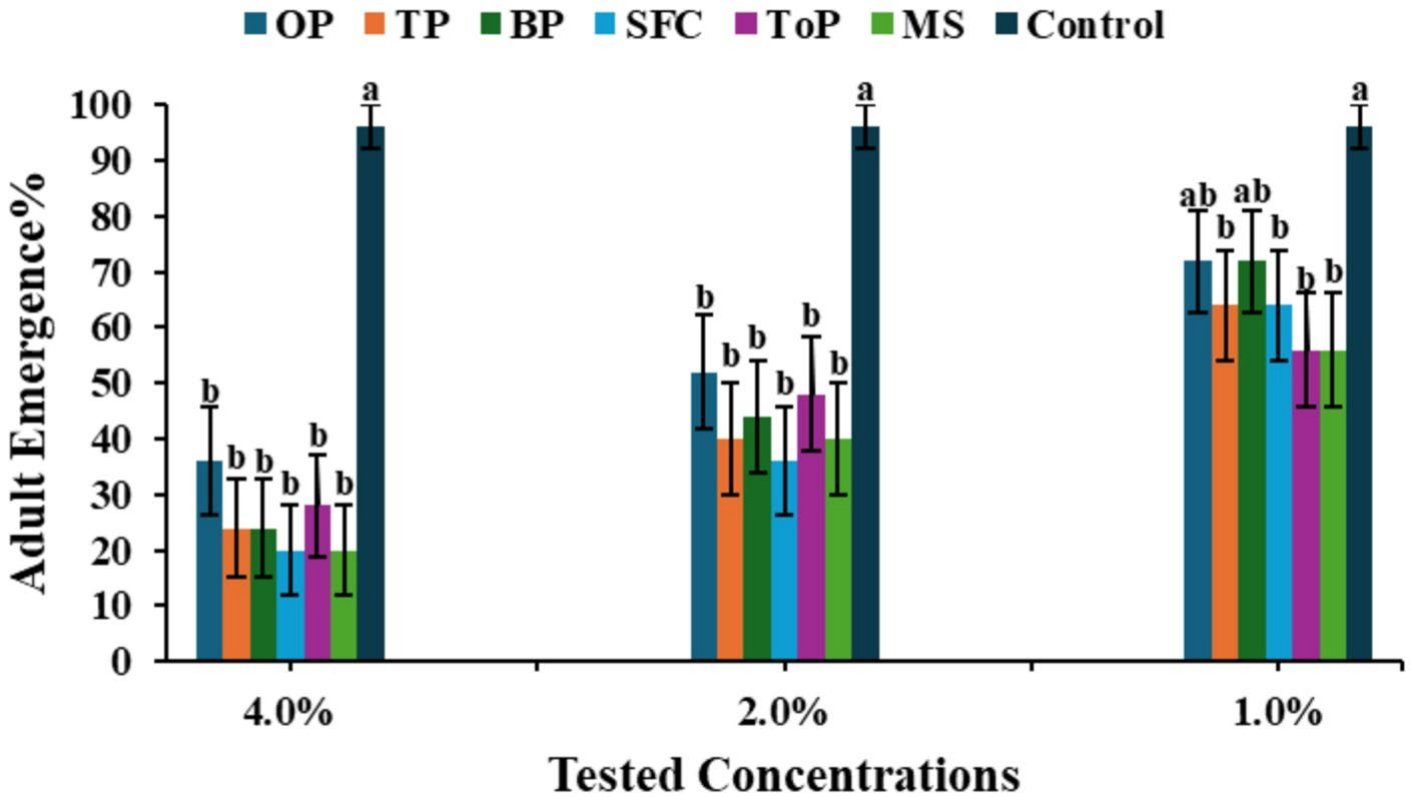
Effect of *Geomyces* sp. MORSY-27 extracts after growing on different agro-industrial residues [orange pomace (OP), taro pomace (TP), banana pomace (BP), sunflower cake (SFC), tomato pomace (ToP), and molokhia stalk (MS)], on the adult emergence % of *A. ipsilon* treated at different concentrations. Mean (± SE) values with different letters within the same concentration are significantly different (*P* ≤ 0.05, ANOVA, Duncan’s test).

Regarding larval mortality, the highest mortality percentages occurred at the 4.0% concentration, with the SFC extract exhibiting the strongest effect, reaching over 70%, followed by TP and MS extracts. Mortality decreased at 2.0% and 1.0%, yet treated larvae still showed significantly higher mortality than the control, which exhibited a minimal mortality rate (Fig. 4a). A similar pattern was observed for pupal mortality, which increased with higher concentrations of the extract. At 4.0%, the SFC and TP extracts resulted in the largest pupal mortality, while no mortality occurred in the control. Although mortality decreased at 2.0% and 1.0%, it remained consistently higher than in untreated larvae (Fig. 4b). The effect of *Geomyces* sp. MORSY-27 extracts on adult emergence of *A. ipsilon* is presented in Fig. 5. Adult emergence was significantly reduced at all tested concentrations, with effects more pronounced at higher concentrations than in the control. At the highest concentration, the SFC extract resulted in the lowest emergence rate (15%), followed by TP and MS extracts, whereas the control group exhibited the highest emergence rate of 95%.

**Fig. 6:**
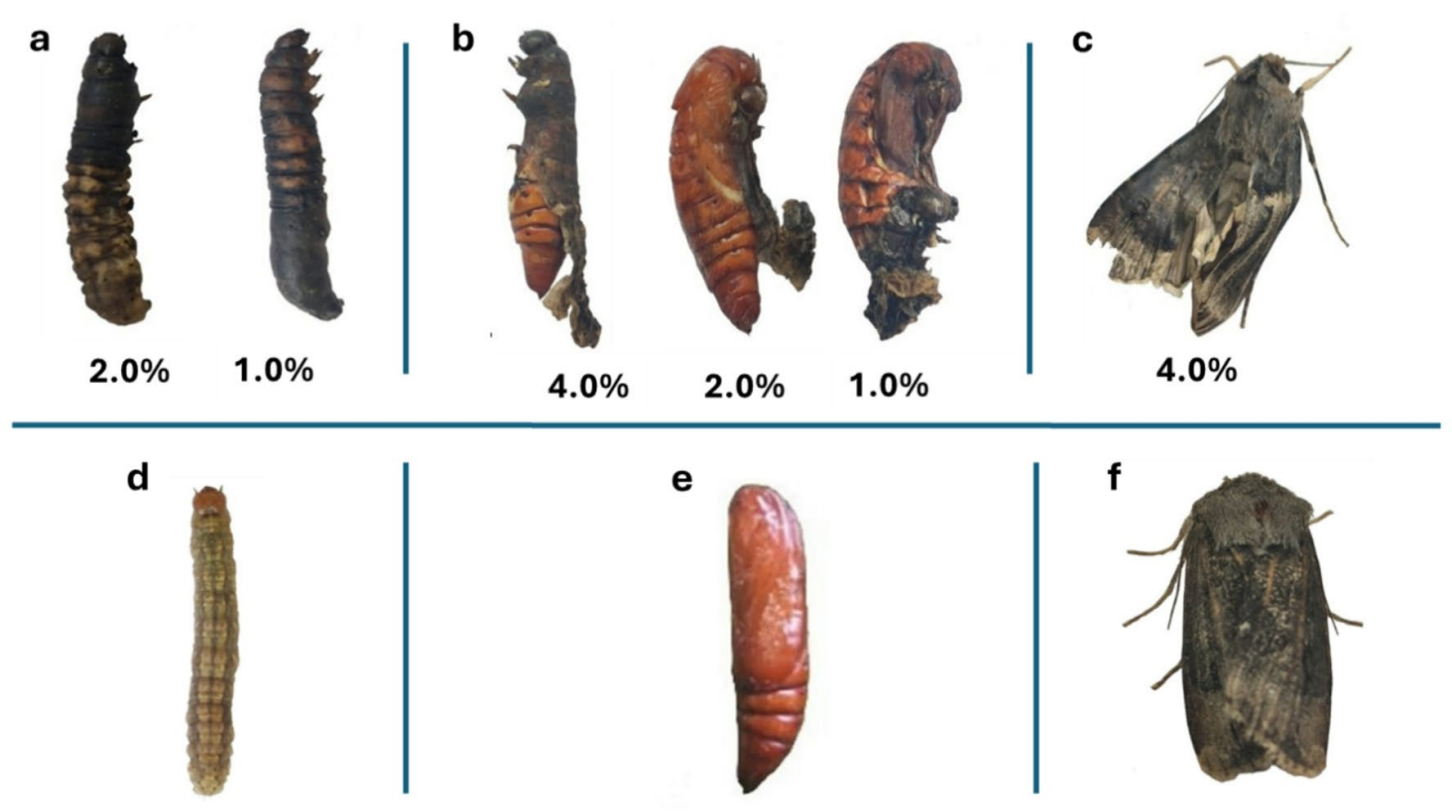
Morphological abnormalities in *A. ipsilon* induced by *Geomyces* sp. MORSY-27 extract grown on sunflower cake (SFC): treated larva **(a)**, pupa **(b)**, and adult moth stage **(c)**, compared with untreated control larva **(d)**, pupa **(e)**, and adult moth **(f)**.

Morphological observations (Fig. 6) revealed that the extract treatments exhibited insect growth regulatory activities against *A. ipsilon*, producing several developmental abnormalities in larvae, pupae, and adults (Fig. 6a, b, c), with the most pronounced effects observed in the SFC extract treatment. *Geomyces* sp. MORSY-27 inhibited normal molting, preventing larvae from completing ecdysis (Fig. 6c) compared with untreated larvae (Fig. 6d), and induced the formation of larval–pupal intermediates (Fig. 6b) instead of normal pupae (Fig. 6e). Additional symptoms included pupae failing to shed their larval or pupal cuticle. Malformed adults exhibited wrinkled wings and incomplete eclosion, in contrast to the normal morphology of untreated insects (Fig. 6f).

#### Acaricidal activity of geomyces sp. MORSY-27

**Fig. 7:**
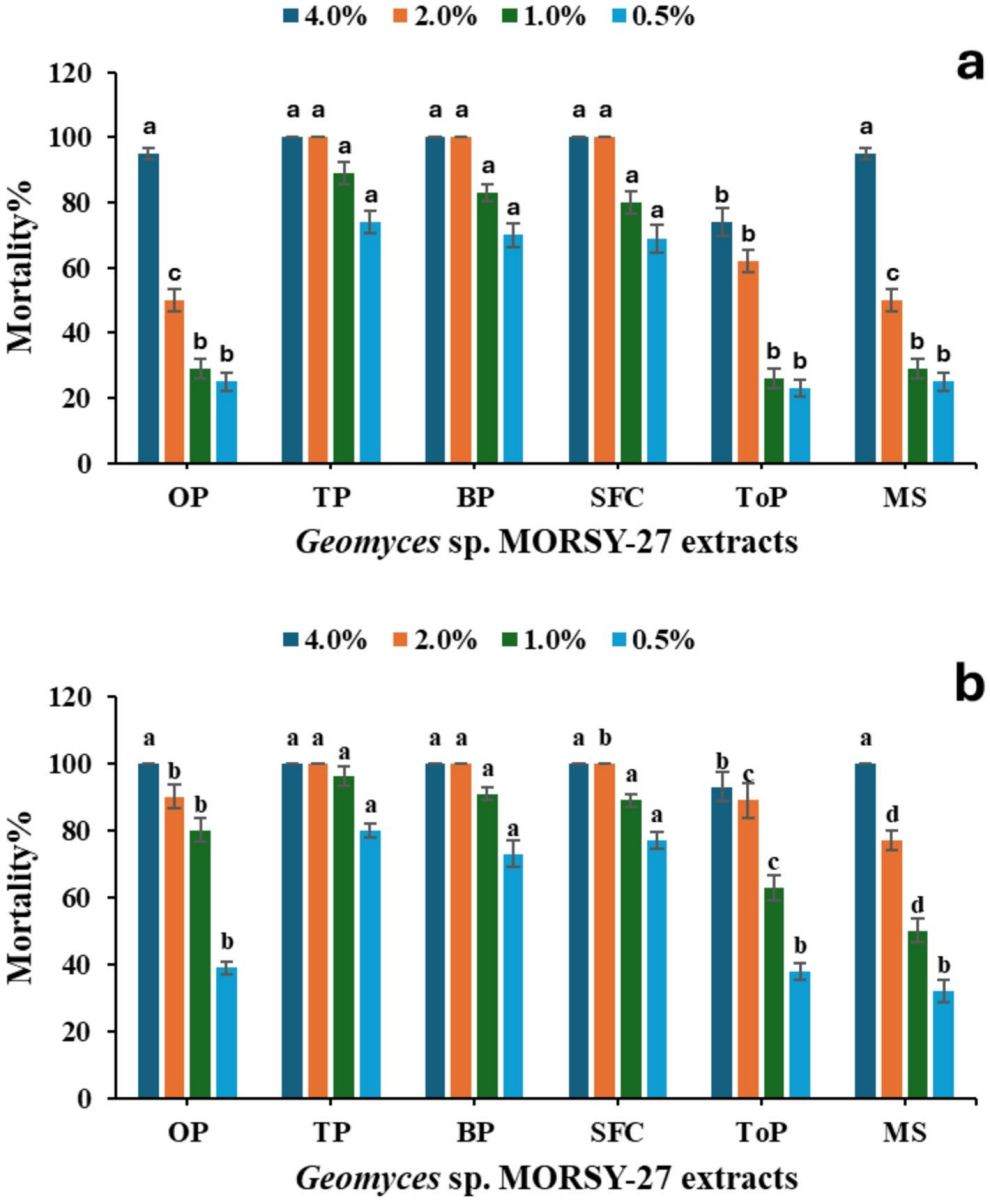
Toxicity of *Geomyces* sp. MORSY-27 extracts, produced on different agro-industrial residues [orange pomace (OP), taro pomace (TP), banana pomace (BP), sunflower cake(SFC), tomato pomace (ToP), and molokhia stalk (MS)], against *T. urticae* females at a: 24 h and b: 96 h post-exposure. Mean (± SE) values with different letters within the same concentration are significantly different (*P* ≤ 0.05, ANOVA, Duncan’s test).

The impacts of *Geomyces* sp. MORSY-27 extracts on *T. urticae* females are illustrated in Fig. 7. All extracts caused significant mortality (*P* ≤ 0.05), with efficacy increasing alongside concentration and exposure time. After 24 h, TP, BP, and SFC extracts at 4.0% and 2.0% resulted in the highest mortality (approaching 100%), whereas the 0.5% concentration consistently showed the lowest mortality rates (≤ 23%) for the other extracts. After 96 h, cumulative mortality reached 100% for TP, BP, and SFC extracts at the higher concentrations (4.0% and 2.0%), reflecting the time-dependent toxic effect. Therefore, the most instructive differences among treatments were observed at lower concentrations (1.0% and 0.5%). At these concentrations, OP and MS extracts exhibited the least toxicity, while TP, BP, and SFC extracts consistently caused the highest mortality. Additionally, mortality at 96 h was markedly higher than at 24 h for all extracts, indicating that their toxic action continues with prolonged exposure. Overall, *T. urticae* female mortality increased significantly with increasing extract concentrations and exposure times. The extracts of TP, BP, and SFC exhibited the highest acaricidal activity, with no significant differences among them at the same concentrations. In contrast, MS and OP extracts recorded the lowest mortality levels, particularly at 0.5% and 1%. After 96 h, mortality approached 100% for most extracts at 4%, highlighting their strong time-dependent toxicity.

**Fig. 8:**
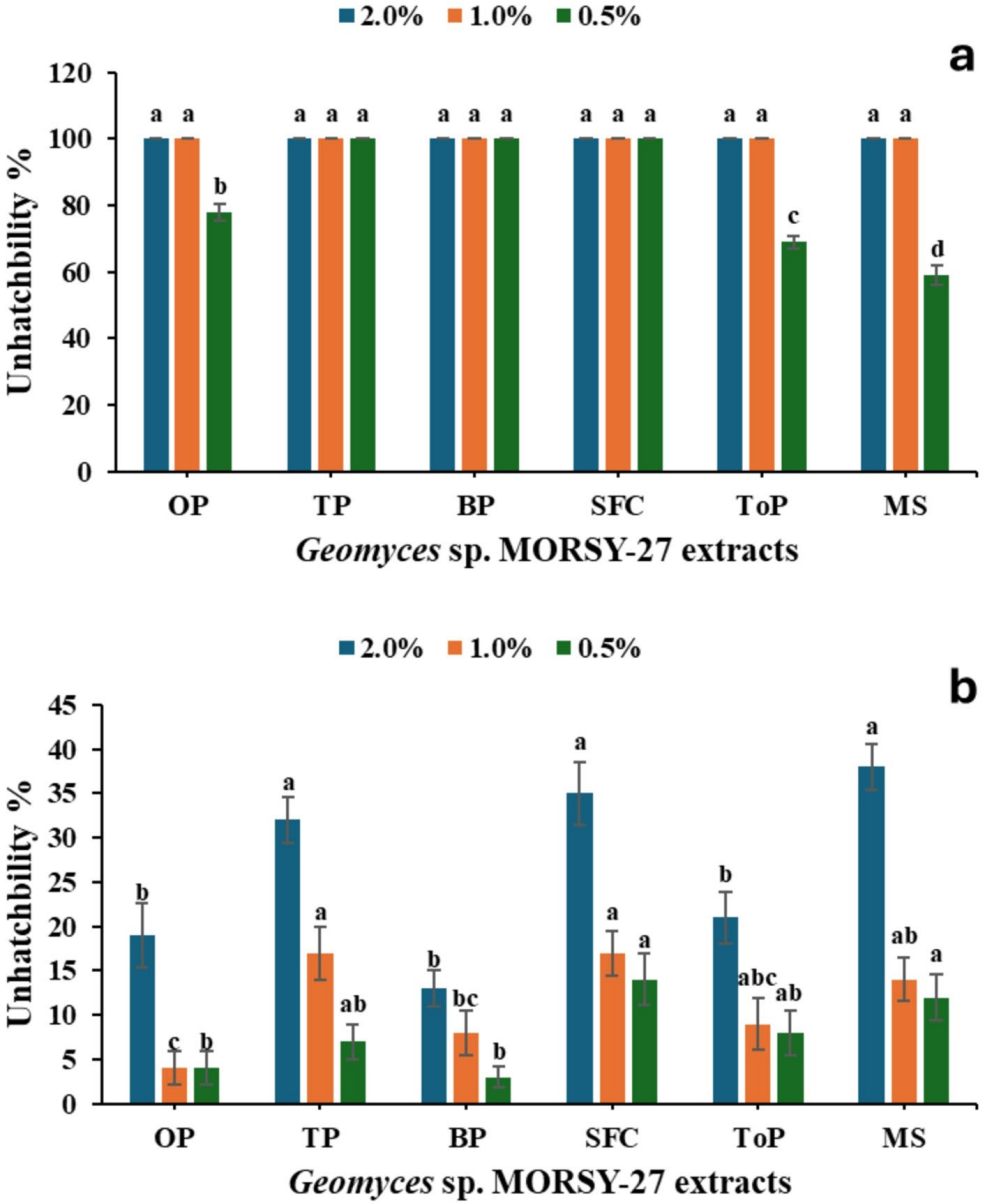
Ovicidal activity of *Geomyces* sp. MORSY-27 extracts against *T. urticae*. **(a)** Un-hatchability (%) of eggs laid by females previously treated with the different extracts. **(b)** Unhatchability (%) of eggs directly exposed to the extracts. Mean (± SE) values with different letters within the same concentration are significantly different (*P* ≤ 0.05, ANOVA, Duncan’s test).

The impact of *Geomyces* sp. MORSY-27 extracts on *T. urticae* eggs is shown in Fig. 8. As illustrated in Fig. 8a, exposure of females to the extracts prior to oviposition significantly reduced egg hatchability. Complete inhibition of hatching (100% unhatched eggs) was recorded in TP, BP, and SFC treatments at all tested concentrations. In contrast, OP and MS extracts were less effective, particularly at 0.5%, where unhatched rates significantly decreased to 85–90%. In contrast, direct exposure of *T. urticae* eggs to the extracts (Fig. 8b) produced considerably lower ovicidal effects compared to pre-oviposition exposure. A significant concentration-dependent reduction in hatchability was observed (*P* < 0.001), but the maximum inhibition did not exceed 35–38%, as observed with SFC and MS extracts at 2.0%. Treatment with OP and BP extracts showed little effect across all concentrations, especially at 0.5%.

### Compounds identified by Gas-Chromatography-Mass spectroscopy in *Geomyces* sp. MORSY-27 extract

**Fig. 9:**
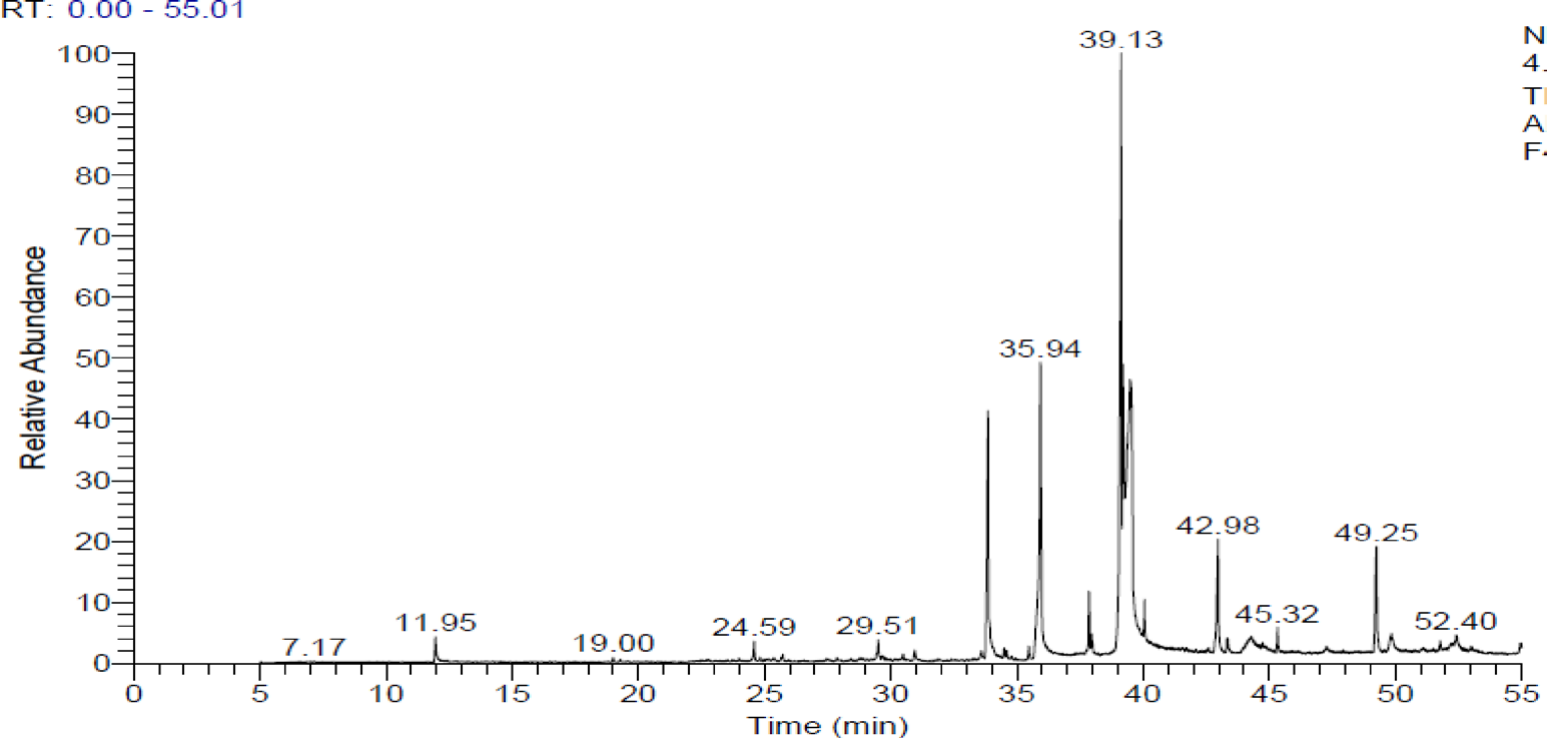
GC-MS Chromatogram of volatile organic compounds detected in the extract of *Geomyces* sp. MORSY-27 grown on sunflower cake (SFC).

As shown in Table S3 and Figs. 9, S2, GC–MS analysis of the crude extract from *Geomyces* sp. MORSY-27, grown on SFC medium, revealed the presence of numerous bioactive secondary metabolites. A total of 39 compounds were identified, including nine predominant metabolites with notable biological activities. The primary compound detected was ethyl 9-cis,11-trans-octadecadienoate, followed by 9,12-octadecadienoic acid (Z, Z) (α-linoleic acid), and N-didehydrohexacarboxyl-2,4,5-trimethylpiperazine. Other major constituents included hexadecanoic acid, ethyl ester (11.54%), 9-octadecenoic acid (Z)-, ethyl ester (6.31%), 13-docosenamide (Z)- (5.16%), 9-octadecenamide (4.93%), methyl 9-cis,11-trans-octadecadienoate (2.33%), and hexadecanamide (1.33%). Additionally, several minor metabolites (0.11–1.14%) were also detected, including 1-propyl-2-methyl-7-methoxy-pyrido[3,4-b]indole, methyl hexadecanoate, heptadecane, stigmasta-5,22-dien-3-ol, and quercetin derivatives. Overall, the GC–MS profile indicates that *Geomyces* sp. MORSY-27 produces a chemically diverse mixture of bioactive fatty acid esters, amides, and alkaloids with strong potential for biocontrol applications (Fig. 9).

## Discussion

The current study shows that endophytic fungi isolated from healthy wheat roots represent a promising source of extracellular enzymes and bioactive secondary metabolites with potential pesticidal activity. Among all isolates collected from the Nile Delta, Middle, and Upper Egypt, MORSY-27 proved to be the most potent, exhibiting the highest levels of metabolite production, enzymatic activity, and biological control effectiveness against *A. ipsilon* and *T. urticae*. These results align with earlier research indicating that endophytic fungi remain an underutilized reservoir of metabolites that enhance host defense and can serve as effective biocontrol agents^55^. Endophytic fungi, which colonize internal plant tissues, play a vital role in agricultural sustainability. They provide numerous benefits to their host plants, including enhanced resistance to herbivores and pests, increased competitiveness, and improved tolerance to abiotic stresses such as heavy metal contamination and high salinity. Moreover, these fungi can influence both the yield and quality of their host plants^56^. Globally, over 50,000 plant species are considered edible, yet only four—wheat, rice, maize, and potato—serve as staple foods for nearly five billion people^57^. Beyond their nutritional significance, these staple crops also harbor rich, diverse endophytic fungal communities that contribute to plant health and resilience. Among the four, wheat hosts the greatest diversity, with 157 endophytic fungal species identified, underscoring its potential as a reservoir of biologically valuable microorganisms.

Solid-state fermentation (SSF) parameters exert a decisive influence not only on fungal growth and enzyme secretion but also on the biosynthesis of pesticidal secondary metabolites, such as phenolics, alkaloids, terpenoids, fatty acids, and cuticle-degrading enzymes, which collectively determine pesticidal efficacy. Moisture content governs water activity and nutrient diffusion, thereby modulating metabolic pathways responsible for synthesizing bioactive compounds; intermediate moisture levels typically enhance phenolic and terpenoid formation by maintaining adequate aeration while preventing substrate compaction^17,18^. Temperature likewise affects the expression of biosynthetic gene clusters linked to secondary metabolite production, with moderate thermic conditions (25–30 °C for most filamentous fungi) stimulating oxidative metabolic routes that generate pesticidal alkaloids, flavonoids, and fatty acids, while excessive heat suppresses secondary metabolism due to thermal stress^19^. The initial substrate pH and its microenvironmental shifts during fermentation also shape the chemical profile of bioactive metabolites, as specific classes, such as cuticle-active lipases, proteases, and chitinases, exhibit narrow pH optima and are downregulated outside their optimal ranges^20^. Substrate particle size further modulates oxygen availability and surface area, influencing the balance between primary growth and secondary metabolite synthesis; intermediate particle sizes promote sufficient gas exchange and nutrient accessibility, thereby enhancing the accumulation of bioactive pesticidal compounds^58^. Aeration is particularly critical in SSF because many bioactive metabolites, including oxidative phenolics and unsaturated fatty acids, require oxygen-dependent enzymatic steps; improved bed porosity and aeration enhance fungal respiration, heat dissipation, and ultimately the synthesis of potent pesticidal metabolites^18^.

The combination of endophytic fungi and SSF represents a strategically advantageous approach for producing pesticidal secondary metabolites. SSF conditions, characterized by low moisture, natural aeration, and nutrient heterogeneity, stimulate fungal secondary metabolism and support higher levels of bioactive compounds than liquid fermentation^17^. The use of agro-industrial residues not only reduces production costs but also provides nutrient-rich substrates that enhance metabolite biosynthesis while contributing to sustainable waste valorization^18^. Moreover, SSF processes are easier to scale, require minimal energy input, and preserve the micro-environmental cues essential for metabolite formation in endophytes^19^. These combined advantages position SSF-driven endophytic fungal fermentation as an efficient, economical, and environmentally responsible platform for producing potent bioinsecticides.

According to the United States Environmental Protection Agency (EPA)^59^, biopesticides are classified into three main categories: plant-incorporated protectants, biochemical pesticides (natural compounds), and microbial pesticides, which utilize microorganisms or their metabolites to manage pests. In this study, the productivity of key microbial pesticide components, including phytochemicals (alkaloids, phenolics, terpenoids, and flavonoids), cuticle-degrading enzymes (chitinases, lipases, and proteases), and fatty acids, was assessed in extracts of root endophytic fungi cultivated on agro-industrial wastes. These bioactive compounds are known to play a critical role in integrated pest management (IPM) by enhancing the biological control potential of microbial agents. The SSF process had a pronounced effect on secondary metabolite production, with sunflower cake (SFC) and taro pomace (TP) supporting the highest yields of phytochemicals and enzymes. Optimal production occurred at 20 °C and 55% IMC, conditions that enhanced fungal metabolism and oxygen availability. These findings corroborate previous reports highlighting SSF as a cost-effective biotechnological approach for valorizing agro-industrial residues into high-value bioproducts^26,60^. The elevated synthesis of chitinase, protease, and lipase in *Geomyces* sp. MORSY-27 is particularly relevant, as these enzymes play a pivotal role in the degradation of pest cuticles. The synergistic action of lipases, proteases, and chitinases facilitates cuticle penetration and infection, ultimately leading to developmental disruption or mortality^61^. Furthermore, the upregulation of antioxidant enzymes such as superoxide dismutase and glutathione peroxidase indicates the activation of protective mechanisms that enhance fungal stress tolerance and support secondary metabolite biosynthesis^62^.

Prior research has shown that lipases and other enzymes produced by entomopathogenic fungi play a role in insect pathogenesis. For instance, *B. bassiana* demonstrated significant lipase activity associated with pronounced virulence against third-instar *Pieris brassicae* larvae^63^. *Mucor hiemalis* exhibited toxicity in *Bradysia odoriphaga* larvae comparable to that of chemical pesticides, decreasing food consumption and lowering total protein, lipid, and carbohydrate levels, while inhibiting essential digestive enzymes, including protease, α-amylase, lipase, and cellulase. This shows how fungal infections can have many different biochemical effects^64^.

This study showed that extracts of *Geomyces* sp. MORSY-27, cultivated on various agro-industrial residues, significantly affected the duration of the larval and pupal stages of *A. ipsilon*. These findings are consistent with earlier research demonstrating that fungal metabolites can disrupt insect development and growth by reducing nutrient absorption, lowering feeding efficiency, or damaging the insect nervous system^65^. A notable decline in adult emergence was also observed in treated groups, particularly at higher extract concentrations. These results are consistent with the work of Akutse et al.^66^and Martinuz et al.^67^, who confirmed that fungal endophytes impacted the physiology of herbivorous insects, thereby protecting their host plants by slowing insect development, reducing growth rates, and decreasing survival and oviposition. Similarly, cauliflower plants inoculated with different endophytic fungi have been tested for their insecticidal effects against *Spodoptera litura* (Fabricius) (Lepidoptera: Noctuidae). The inoculated plants induced significant larval mortality, reduced adult emergence, extended developmental duration, and negatively affected the reproductive potential of *S. litura*^68^.

Malformations such as shriveled wings, darkened and shrunken pupae, and incomplete eclosion reflect disturbances in cuticle formation and hormonal regulation during the pupal–adult transition. Our results are consistent with those of Al-Ani et al.^69^, who confirmed the biological efficacy of alkaloids against agricultural pests, including insects, fungi, bacteria, and other pests. Owing to their chemical diversity, alkaloids exert multiple modes of action, functioning as growth regulators, toxic agents, or repellents. Additionally, the insecticidal mechanisms of alkaloids, phenolics, flavonoids, terpenoids, and fatty acids produced by fungi are diverse, making them valuable candidates for biopesticide development. Alkaloids, in particular, operate through neurotoxicity, enzyme inhibition, and feeding deterrence while offering key advantages such as biodegradability, target specificity, and a lower likelihood of resistance development^70^. It was reported that sabadilla alkaloids are effective insecticides that induce paralysis by disrupting nerve cell membranes and interfering with sodium channel function, exhibiting a mode of action similar to that of pyrethroids^71^.

The insecticidal effects of phytochemical compounds were also demonstrated against the larval stage of *S. littoralis*^72^. Feeding larvae with black pepper fruit extracts resulted in 100% mortality, and the treatment significantly prolonged both larval and pupal developmental periods. These effects are attributed to the presence of bioactive phytochemicals in the extract, particularly the alkaloid piperine. Terpenoids also play a major defensive role, functioning as natural repellents, toxins, and growth inhibitors, key components of plant protection strategies. Terpenoids have been widely recognized as eco-friendly pesticides and bio-drugs that mitigate the harmful effects of chemical agents^52,73^. Mono- and sesquiterpenes from *Artemisia nakaii* essential oils, for instance, show potential as botanical pesticides against *S. litura*^74^. Plants under herbivore attack often deploy terpenoids to disrupt pest development, feeding, and survival. Terpenoids have been reported to exhibit antifeeding activity against several stored-product insect pests, including *Sitophilus granarius*, *Trogoderma granarium*, and *Tribolium confusum*^75^. Phenolic compounds, mimicking natural plant defenses, interfere with insect nervous and metabolic systems, inhibit molting enzymes, and reduce feeding and reproduction. Likewise, flavonoids and isoflavonoids exhibit potent insecticidal and antifeedant effects, providing safe, sustainable alternatives to synthetic pesticides^76^.

Endophytic fungi can markedly reduce pest populations and fitness. Certain fungal isolates enhanced maize defense against *S. frugiperda*, reducing oviposition, delaying pupation, and reducing adult emergence relative to untreated controls^77^. Larval mortality peaked during the second larval and pupal stages when insects fed on leaves fully colonized by endophytes. Similarly, Russo et al.^78^demonstrated that the endophytic fungus *B. bassiana* negatively affected *S. frugiperda* growth and reproduction, significantly reducing larval and pupal survival and prolonging developmental duration. The isolates of *B. bassiana* exhibited significant insecticidal efficacy, resulting in 71.7–98.3% and 60–100% mortality rates in nymphs of *Bemisia tabaci* and *Trialeurodes vaporariorum*, respectively, while adult mortality varied from 58 to 94.3% (*B. tabaci*) to 59–95.4% (*T. vaporariorum*). These high death rates were associated with higher levels of enzymes, including chitinase, lipase, and protease. This suggests that extracellular enzymes are very important in making fungi more dangerous to both nymphs and adults of whiteflies^79^. These findings support the role of endophytic fungi as effective biological control agents by disrupting insect growth and reproduction. Additionally, certain fatty acids naturally produced by entomopathogenic fungi exhibit strong insecticidal and repellent activities against multiple insect species while remaining safe for humans and the environment^80^. Collectively, these fungal-derived metabolites represent a sustainable and multifaceted approach to biological pest control.

In this study, extracts of *Geomyces* sp. MORSY-27 exhibited significant adulticidal and ovicidal activity against *T. urticae*. The pronounced toxicity, particularly at higher concentrations, reflects the abundance and potency of metabolites produced by this fungus. Among the tested treatments, sunflower cake (SFC) extract consistently demonstrated the highest efficacy across bioassays, highlighting its potential as a promising myco-acaricide for *T. urticae*. These findings align with previous biochemical characterization of the MORSY-27 extract, which demonstrated the strong production of cuticle-degrading enzymes, including proteases, chitinases, and lipases^81^. These enzymes facilitate the disruption of the mite cuticle, enhancing the penetration and delivery of toxic metabolites into internal tissues^82^. In parallel, the diverse phytochemicals and fatty acids produced by this isolate exhibit well-documented insecticidal and acaricidal activities, including membrane disruption and interference with cellular homeostasis^83^. Interestingly, our findings showed near-complete inhibition of egg hatchability following pre-oviposition exposure, especially with SFC, TP, and BP extracts. Unlike the entomopathogenic fungus *B. bassiana*, which has been reported to have negligible effects on egg viability when adults are treated, showing no ovicidal or transovarial impact^84^. This pronounced effect may result from interference with female reproductive physiology or the transovarial transfer of toxic compounds that impair embryogenesis, consistent with previous observations on entomopathogenic fungi affecting spider mite fecundity and offspring viability^85^. Conversely, in this study, eggs directly exposed to the extracts exhibited lower susceptibility, which could be explained by the chorion’s limited permeability, acting as a protective barrier to metabolite penetration. Despite this, the moderate yet significant inhibition caused by SFC and MS extracts at higher concentrations indicates that the extracts can still compromise egg development when dose and exposure conditions are optimized.

Gas chromatography–mass spectrometry (GC-MS) analysis confirmed the presence of 39 metabolites in the *Geomyces* sp. MORSY-27 extract, several of which have been previously associated with insecticidal, larvicidal, and antifungal activities. Predominant compounds included ethyl 9-cis,11-trans-octadecadienoate; 9, 12-octadecadienoic acid (Z, Z); hexadecanoic acid ethyl ester; and 13-docosenamide (Z). These fatty acid derivatives and amides act by disrupting membranes, inhibiting chitin synthesis, and interfering with insect hormonal balance^86,87^. The diversity of these compounds underscores the metabolic versatility of *Geomyces* sp. MORSY-27 and its suitability as a candidate for the development of eco-friendly mycopesticides. The biochemical diversity and bio-efficacy of *Geomyces* sp. MORSY-27 positions it as a promising biotechnological agent for integrated pest management (IPM) strategies aimed at reducing dependence on synthetic pesticides.

## Conclusions

This study highlights *Geomyces* sp. MORSY-27 as a promising endophytic fungus capable of producing a diverse array of bioactive metabolites with potent insecticidal and acaricidal properties. The strain was identified through morphological, biochemical, physiological, and molecular analyses and exhibited high biosynthetic potential under solid-state fermentation using low-cost agro-industrial residues. Among the substrates tested, sunflower cake and taro pomace were most effective in enhancing the production of phytochemicals (phenolics, flavonoids, alkaloids, and terpenoids) and enzymes (chitinase, protease, and lipase), particularly under optimized conditions of 20 °C and 55% initial moisture content. GC–MS analysis confirmed 39 secondary metabolites in *Geomyces* sp. MORSY-27, predominantly fatty acid esters, amides, and alkaloids, which are known for their antimicrobial and insecticidal activities. Extracts from this strain markedly prolonged larval and pupal development, increased mortality, and induced severe deformities in *A. ipsilon*, suggesting disruption of hormonal regulation and cuticle formation. Extracts of *Geomyces* sp. MORSY-27 exhibited strong, dose- and time-dependent acaricidal activity against *T. urticae*, achieving up to 100% mortality and complete inhibition of egg hatch, highlighting its potential as an eco-friendly pest control agent. These findings demonstrate the broad-spectrum biocontrol potential of *Geomyces* sp. MORSY-27, offering a sustainable and cost-effective alternative to chemical pesticides. To promote practical application, future studies should focus on broader dose-time assessments and comparisons with established pesticidal products. Moreover, future research should focus on isolating the active compounds, assessing their safety, elucidating their molecular mechanisms, and evaluating field efficacy to support their application in integrated pest management (IPM) programs.

## Supplementary Information

Below is the link to the electronic supplementary material.

Supplementary Material 1

## Data Availability

All data generated or analyzed during this study are included in this published article and supplementary materials. Molecular identification of strain MORSY-27 using ITS1/ITS4 primers generated a 534 bp rDNA-ITS fragment, which was subsequently sequenced and deposited in NCBI GenBank under the assigned accession number PX588620 (https://www.ncbi.nlm.nih.gov/nuccore/PX588620.1).
