# Supplementary material for: Sustainable production of insecticidal and acaricidal metabolites by endophytic fungi using solid-state fermentation
Source: Sci Rep. 2026 Apr 3;16:11356. doi: 10.1038/s41598-026-40413-w (PMC13049106; doi:10.1038/s41598-026-40413-w)
Supplement: Supplementary file 1 — Supplementary Material 1 [file 41598_2026_40413_MOESM1_ESM.docx]

**
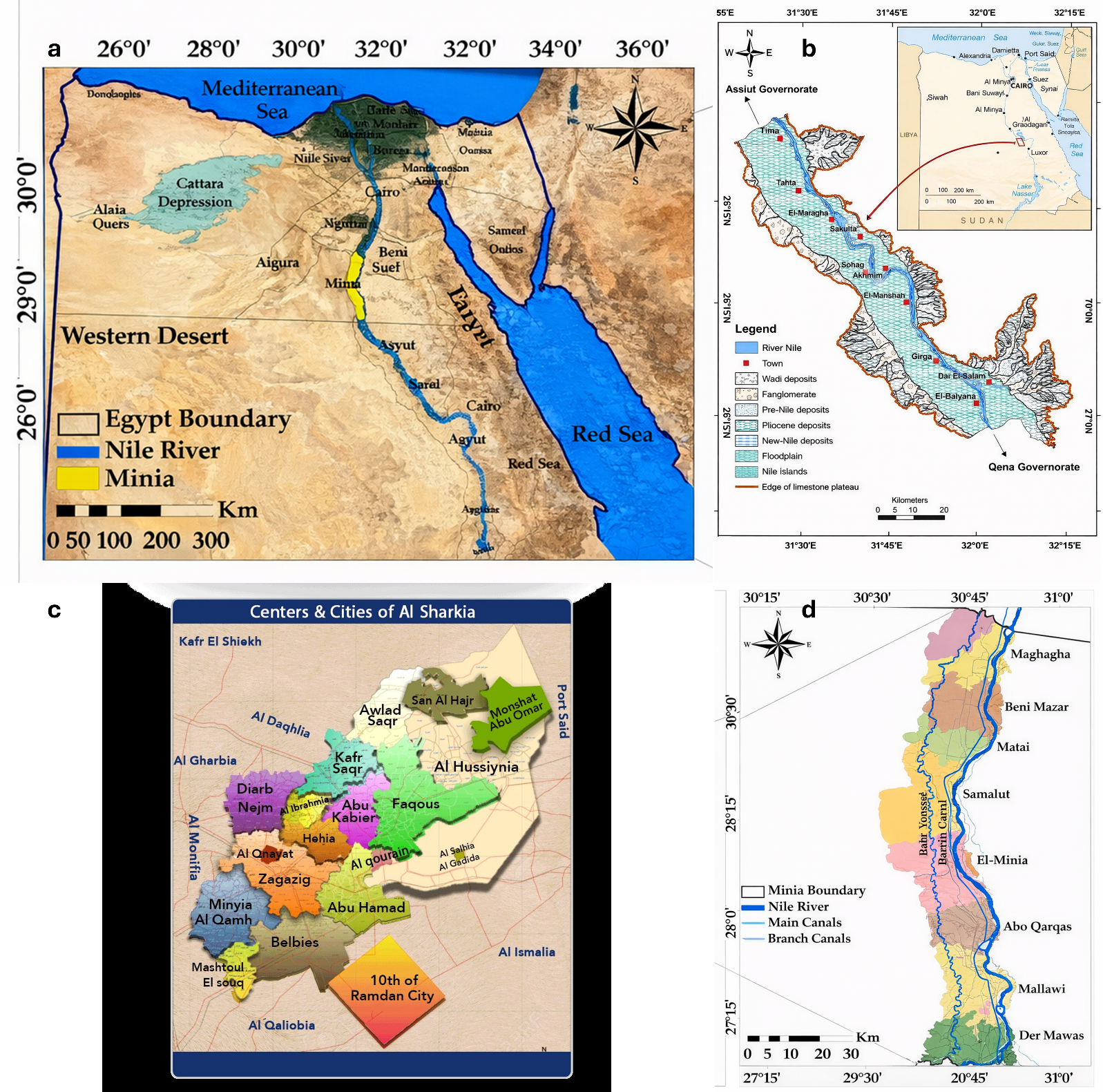
**

**Fig. S1.** Location map of the study’s sampling sites (a: Governorates of Egypt, b: Sohag, c: Sharqia, d: Minya- Google map).

**(A, 23.90%) (B, 17.26%) (C, 11.63 %)**

**(D, area % 11.54) (E, area % 6.31) (F, area % 5.16)**

**(G, area % 4.93) (H, area % 2.33) (I, area % 1.33)**

**Fig. S2.** GC-MS Chromatograms of the predominant compounds Ethyl 9-cis,11-trans-octadecadienoate (A); 9, 12-Octadecadienoic acid (Z, Z) - (CAS) (B); N-Didehydrohexacarbo xyl-2,4,5-trimethylpipe razine (C); Hexadecanoic acid, ethyl ester (D); 9-Octadecenoic acid (Z)-, ethyl ester (CAS) (E); 13-Docosenamide, (Z)- (F); 9-Octadecenamide (CAS) (G); Methyl 9-cis,11-trans-octadeca dienoate (H); and Hexadecanamide (CAS) (I)

**Table S1:** Evaluation of the ability of wheat-derived endophytic fungi from different regions to produce pesticidal metabolites using a taro pomace (TP) medium

| **Isolate** | **Pesticidal metabolites of endophytic fungi** | | | | | | | |
| --- | --- | --- | --- | --- | --- | --- | --- | --- |
|  | **Phytochemicals (mg/gds)** | | | | **Cell wall and cuticle hydrolytic enzymes (U/gds)** | | | **Total fatty acids (mg/gds)** |
|  | **Alkaloid** | **Phenolic** | **Terpenoid** | **Flavonoid** | **Chitinase** | **Lipase** | **Protease** |  |
| **Delta region**  MORSY-1  MORSY-2  MORSY-3  MORSY-4  MORSY-5  MORSY-6  MORSY-7  MORSY-8  MORSY9  MORSY-10  MORSY-11  MORSY-12  MORSY-13  MORSY-14  MORSY-15  **Middle Egypt**  MORSY-16  MORSY-17  MORSY-18  MORSY-19  MORSY-20  MORSY-21  MORSY-22  MORSY-23  MORSY-24  MORSY-25  MORSY-26  **Upper Egypt**  MORSY-27  MORSY-28  MORSY-29  MORSY-30  MORSY-31  MORSY-32  MORSY-33  MORSY-34  MORSY-35  MORSY-36  MORSY-37  MORSY-38 | 50.72 ± 3.88  23.16 ± 1.92  18.50 ± 1.60  42.71 ± 2.99  36.19 ± 2.83  27.50 ± 1.98  19.27 ± 1.75  38.17 ± 2.89  23.90 ± 1.95  54.74 ± 4.10  48.21 ± 3.70  25.40 ± 2.06  40.56 ± 3.14  48.70 ± 3.55  51.20 ± 3.81  40.12 ± 2.91  26.16 ± 2.13  33.90 ± 2.74  50.36 ± 3.85  24.22 ± 2.12  35.80 ± 2.87  40.27 ± 3.12  52.13 ± 3.90  46.25 ± 3.57  30.60 ± 2.69  57.16 ± 4.52  61.15 ± 4.75  42.25 ± 3.25  29.82 ± 2.13  51.90 ± 3.86  30.57 ± 2.69  44.00 ± 3.40  28.30 ± 2.08  35.27 ± 2.87  22.89 ± 1.79  59.20 ± 4.70  30.11 ± 2.66  25.80 ± 2.15 | 115.70 ± 5.48  230.29 ± 8.00  174.52 ± 6.81  200.35 ± 7.64  149.25 ± 6.37  118.76 ± 5.55  172.30 ± 6.60  159.18 ± 6.26  225.18 ± 7.97  180.20 ± 6.75  240.17 ± 7.18  160.85 ± 6.37  135.00 ± 6.10  221.15 ± 7.93  216.42 ± 7.82  233.35 ± 8.14  127.40 ± 5.29  212.65 ± 7.58  175.11 ± 6.75  128.25 ± 5.34  241.50 ± 8.16  172.13 ± 6.70  200.44 ± 7.67  187.10 ± 7.00  212.33 ± 8.74  225.70 ± 7.85  245.42 ± 8.90  135.18 ± 5.47  190.00 ± 7.03  234.52 ± 8.12  190.31 ± 7.02  176.12 ± 6.80  85.60 ± 4.59  99.12 ± 4.86  113.00 ± 5.09  180.42 ± 6.83  200.72 ±7.63  215.30 ± 7.70 | 56.47 ± 4.57  42.30 ± 3.25  31.65 ± 2.46  30.12 ± 2.35  49.00 ± 3.86  50.71 ± 4.00  45.28 ± 4.11  46.31 ± 4.20  55.42 ± 4.28  33.50 ± 2.50  28.26 ± 2.17  37.50 ± 2.80  40.21 ± 3.12  44.25 ± 3.80  60.13 ± 4.69  30.71 ± 2.36  45.52 ± 4.16  17.94 ± 1.46  20.28 ± 1.53  38.56 ± 2.87  40.70 ± 3.14  29.46 ± 2.00  37.18 ± 2.92  25.74 ± 1.80  32.50 ± 2.55  25.76 ± 1.82  69.18 ± 5.73  43.00 ± 4.00  30.52 ± 2.34  29.10 ± 2.29  36.15 ± 2.88  48.13 ± 4.17  37.64 ± 3.00  50.82 ± 4.39  30.11 ± 2.31  45.70 ± 4.22  34.06 ± 2.50  39.80 ± 3.06 | 83.60 ± 6.25  72.24 ± 5.46  60.18 ± 4.98  79.00 ± 5.79  66.20 ± 5.13  84.19 ± 6.47  90.50 ± 7.30  70.33 ± 5.25  88.26 ± 6.69  62.21 ± 5.24  71.90 ± 5.30  85.72 ± 6.53  66.17 ± 5.58  59.30 ± 4.92  75.46 ± 5.69  64.50 ± 5.00  86.31 ± 6.76  70.93 ± 5.23  67.50 ± 5.12  82.00 ± 6.50  51.13 ± 3.97  70.56 ± 5.22  90.17 ± 6.98  62.40 ± 5.21  73.51 ± 5.52  56.12 ± 4.28  92.84 ± 7.17  51.49 ± 3.90  45.00 ± 3.67  82.44 ± 6.48  90.13 ± 6.99  65.80 ± 5.18  49.12 ± 3.84  73.80 ± 5.90  85.12 ± 6.70  63.70 ± 5.02  52.41 ± 3.98  70.65 ± 5.24 | 653.14 ± 14.25  293.40 ± 6.85  525.29 ± 11.90  463.42 ± 10.27  619.83 ± 13.70  310.43 ± 7.13  436.92 ± 9.12  421.50 ± 9.83  318.90 ± 7.00  536.54 ± 1200  441.90 ± 9.05  529.50 ± 11.91  245.88 ± 6.25  418.20 ± 9.86  327.58 ± 7.18  530.60 ± 11.93  729.18 ± 16.00  616.90 ± 14.05  440.72 ± 9.03  326.50 ± 7.19  534.26 ± 12.03  319.42 ± 7.00  427.95 ± 8.75  630.40 ± 14.28  235.28 ± 6.45  594.10 ± 13.67  847.65 ± 19.30  626.88 ± 14.11  485.91 ± 10.40  510.26 ± 11.52  478.39 ± 10.28  523.61 ± 11.70  298.84 ± 6.60  540.62 ± 12.05  637.88 ± 14.50  824.76 ± 19.47  590.21 ± 13.60  616.88 ± 15.74 | 261.45 ± 7.89  173.20 ± 5.73  279.20 ± 8.30  79.48 ± 3.00  207.56 ± 6.43  266.40 ± 8.25  189.62 ± 6.00  170.38 ± 5.68  247.50 ± 7.66  87.40 ± 3.29  98.94 ± 3.97  250.53 ± 7.59  113.94 ± 4.50  168.43 ± 5.69  223.51 ± 7.28  140.68 ± 4.84  270.15 ± 7.93  252.28 ± 7.57  176.43 ± 5.71  235.20 ± 7.60  154.90 ± 5.72  260.43 ± 7.76  143.21 ± 5.60  57.96 ± 2.59  74.32 ± 2.87  211.50 ± 7.00  280.31 ± 7.85  63.50 ± 2.71  90.28 ± 3.30  200.16 ± 6.93  75.13 ± 2.90  81.42 ± 3.14  90.13 ± 3.38  132.40 ± 5.52  265.80 ± 7.63  79.64 ± 3.18  101.44 ± 4.31  158.69 ± 5.87 | 995.40 ± 17.06  711.23 ± 12.00  1010.48 ± 17.33  913.50 ± 15.47  844.75 ± 13.90  1000.47 ± 16.28  991.68 ± 16.95  751.70 ± 12.18  1000.12 ± 17.12  866.00 ± 14.47  852.14 ± 14.00  906.28 ± 15.83  720.63 ± 11.87  895.12 ± 14.60  768.38 ± 12.47  916.50 ± 15.13  898.20 ± 14.62  1017.65 ± 16.85  871.32 ± 14.00  890.68 ± 14.61  784.21 ± 13.00  940.56 ± 16.02  675.44 ± 12.48  853.70 ± 15.69  642.60 ± 12.00  813.20 ± 13.97  1020.49 ± 17.45  1009.40 ± 17.30  852.00 ± 14.57  950.74 ± 16.69  715.30 ± 12.00  900.16 ± 15.70  637.22 ± 11.75  991.40 ± 17.21  1005.38 ± 17.98  862.90 ± 16.00  985.21 ± 17.14  965.17 ± 16.83 | 22.56 ± 2.75  19.24 ± 2.61  50.50 ± 4.50  20.18 ± 2.38  47.55 ± 4.37  16.30 ± 2.46  35.27 ± 3.94  17.59 ± 2.23  26.00 ± 3.45  42.08 ± 4.19  30.39 ± 3.20  39.88 ± 3.79  24.50 ± 2.88  27.35 ± 3.38  40.13 ± 3.92  35.75 ± 3.66  24. 38 ± 2.85  39.14 ± 3.73  40.25 ± 3.82  37.42 ± 3.70  46.75 ± 4.16  28.90 ± 3.58  15.84 ± 2.19  21.18 ± 2.65  32.60 ± 3.25  52.37 ± 4.30  57.39 ± 4.34  39.40 ± 3.73  47.12 ± 4.25  24.63 ± 3.04  49.88 ± 4.00  30.11 ± 3.48  26.90 ± 3.27  44.81 ± 3.96  35.40 ± 3.57  49.68 ± 4.13  50.19 ± 4.20  40.13 ± 3.91 |

**Table S2:** Phenotypic and chemotypic characteristics of the selected endophytic isolate MORSY-27

| **Parameter** | **Behavior** |
| --- | --- |
| **Growth characteristics on**  **Potato dextrose agar** | Well growth, good sporulation; superficial colonies, soft and fluffy, 48–52 mm in diameter, upper surface white with light green centre turned to bright yellow; reverse dark brown at the center surrounded by yellowish-brown edge with a regular margin; produces a faint earthy odor, and light green diffusible pigment. |
| **Sabouraud dextrose agar** | Presented good sporulation; velvety colonies, 40–44 mm in diameter; upper side whitish orange turned to brownish beige and dark brown periphery with a regular margin; reverse yellow-brown at the center and pale green at the edges; produced a faint moldy odor; no diffusible pigment detected. |
| **Czapek-Dox agar** | Abundant sporulation; **granular** colonies, 52 to 56 mm in diameter; upper side pastel brown in center and pale orange-yellow in margin, while the reverse is dark brown in center with yellowish brown edge and entire margin; no diffusible pigment detected. |
| **Malt extract agar** | Good growth and sporulation; powdery colonies, 52–64 mm in diameter, flat with raised edge; the front was bright cream with a white edge, and the reverse dark olivaceous. The edge of irregular and smooth waves gave a dull, olive, diffusible pigment and a faint, moldy odor. |
| **Peptone yeast extract agar** | Displayed good growth and sporulation; wrinkled and raised colonies, 55–62 mm; surface grayish olive with green edge; reverse dull brown with regular margin, no odor, and diffused pigment were detected. |
| **Czapek yeast extract agar** | Gave good sporulation; colonies were circular with an entire edge. The upper surface was yellowish-white, turning to a grayish-yellowish-brown, and had a finely powdery texture. The colonies were 45–50 mm in diameter. The reverse was deep green. The fungus produced a diffusible, dark brown pigment, which formed exudates on the surface. |
| **Temperature** | Temperature range 4 - 34 °C, optimum temperature 10.0 to 25 °C, but no growth was observed at 37°C. |
| **pH** | The pH range for growth was 4.0 to 8.0, with an optimum of pH 5.0–5.5. |
| **Microscopic characteristics** | Conidiophores are hyaline, erect, narrow, smooth, and thin-walled, commonly bearing verticils of 3–4 branches; arise directly from the substrate or the aerial hyphae, smooth without septate, 16–20 × 4.5–5.8 µm in diameter, have tree-like sporulating structures, short branched at acute angle, bearing short chains conidia formed at the tips of branched conidiophore. The terminal or lateral conidia are aseptate, smooth, pyriform to clavate with rounded ends and tend to swell slightly during maturation, and their size was 3.5– 8.2× 2.7–5.2 μm. |
| **Biochemical characteristics** | It was able to produce phosphatase, β-β-glucosidase, esterase, lipase, N-acetyl-β-glucosaminidase-galactosidase, β-galactosidase, β-glucoronidase, α-glucosidase, β-glucosidase, proteinases, α-mannosidase, and α-fucosidase. Still, it failed to produce valine arylamidase, cystine arylamidase, leucine arylamidase, and α-chymotrypsin. |

### **Table S3.** GC-MS analysis and volatile active compounds in the *Geomyces* sp. MORSY-27 extract after growing on sunflower cake (SFC) culture

| **Peak No** | **RT** | **Prob.** | **Area %** | **MW** | **Molecular formula** | **Chemical structure** | **Identified compounds** | **Biological activity** |
| --- | --- | --- | --- | --- | --- | --- | --- | --- |
| 1 | 11.95 | 8.77 | 1.03 | 136 | C_10_H_16_ |  | Cyclohexene, 1-methyl-5-(1-methylethenyl)-, (R)- | Antimicrobial, antibiofilm[**1**] |
| 2 | 19.00, 24.59, 24.83, 25.72, 27.87, 28.79, 28.92, 29.68 | 6.14, 7.83, 7.02, 14.28, 7.26,17.87, 30.74, 8.22 | 0.15, 0.74, 0.14,0.36, 0.19,0.23,0.15, 0.19 | 310 | C_22_H_46_ |  | Docosane (CAS) | Antiaging, antifungal- antitumor, larvicidal, antimicrobial, cytotoxic [**2]** |
| 3 | 25.36 | 4.57 | 0.21 | 220 | C_14_H_20_O_2_ |  | Benzo[b]dihydropyran, 6-hydroxy-4, 4, 5, 7, 8-pentamethyl | Antifungal, antioxidant [**3**] |
| 4 | 29.51 | 7.31 | 0.87 | 450 | C_32_H_66_ |  | Docosane, 11-decyl- (CAS) | Antimicrobial, cytotoxic against MCF-7, HePG2 [**4**] |
| 5 | 30.17 | 16.21 | 0.11 | 324 | C_23_H_48_ |  | Heptadecane, 9-hexyl- | Antimicrobial and antifungal agent [**5**] |
| 6 | 30.95 | 14.21 | 0.48 | 218 | C_15_H_22_O |  | 4, 6, 6-Trimethyl-2-(3-methylbuta-1, 3-dienyl)-3-oxatricyclo [5.1.0.0(2, 4)] octane | Antifeeding and Insecticidal [**2**] |
| 7 | 31.89 | 11.68 | 0.17 | 312 | C_20_H_40_O_2_ |  | Octadecanoic acid, ethyl ester (CAS) | Antiviral, antibacterial and antioxidant [**2**] |
| 8 | 33.59 | 7.78 | 0.43 | 336 | C_22_H_40_O_2_ |  | 2H-Pyran, 2-(2-heptadecynyloxy) tetrahydro- (CAS) | - |
| 9 | 33.85 | 67.18 | 11.63 | 222 | C_13_H_22_N_2_O | - | N-Didehydrohexacarboxyl-2, 4, 5-trimethylpiperazine | Antifungal, antiinsect and antioxidant activity[**6**] |
| 10 | 34.49 | 89.12 | 0.41 | 276 | C_17_H_24_O_3_ |  | 7, 9-Di-tert-butyl-1-oxaspiro (4, 5) deca-6, 9-diene -2, 8-dione | - |
| 11 | 34.60 | 35.90 | 0.24 | 270 | C_17_H_34_O_2_ |  | Hexadecanoic acid, methyl ester (CAS) | Antioxidants, hypocholesterolemic, nematicide,Anti-tumoral, anti-microbial, anti-oxidant, decrease blood cholesterol, anti-inflammatory[**7**] |
| 12 | 34.80, 41.54 | 7.94, 11.11 | 0.12, 0.16 | 366 | C_26_H_54_ |  | Octadecane, 3-ethyl-5-(2-ethylbutyl) - (CAS) | Anti-inflammatory, antioxidant, anthelmintic activities [**8**] |
| 13 | 35.47 | 10.32 | 0.54 | 278 | C_16_H_22_O_4_ |  | Dibutyl phthalate | Antibacterial against Gram-positive and negative bacteria, antifungal [**9**] |
| 14 | 35.94 | 58.31 | 11.54 | 284 | C_18_H_36_O_2_ |  | Hexadecanoic acid, ethyl ester | Mosquito larvicidal, chitinase and ecdysone-20 monooxygenase inhibitors, antioxidants, hypocholesterolemic, nematicide, and pesticide [**10**] |
| 15 | 37.86 | 12.64 | 2.33 | 294 | C_19_H_34_O_2_ |  | Methyl 9-cis, 11-trans-octadecadienoate | Antioxidant, anti-cancer, and antimicrobial [**6**] |
| 16 | 37.96 | 5.73 | 0.77 | 296 | C_19_H_36_O_2_ |  | 9-Octadecenoic acid, methyl ester (CAS) | Antioxidants, hypocholesterolemic, nematicide, and pesticide [**1**] |
| 17 | 38.20 | 10.94 | 0.09 | 330 | C_21_H_30_O_3_ |  | Estra-1, 3, 5(10)-triene, 3, 16, 17-trimethoxy-, (16à, 17à)- | - |
| 18 | 39.12 | 44.27 | 23.90 | 308 | C_20_H_36_O_2_ |  | Ethyl 9.cis.,11.trans.-octadecadienoate | Antifeeding, antiinsect, antioxidant, anti-malarial, antifungal [**11**] |
| 19 | 39.21 | 12.45 | 6.31 | 310 | C_20_H_38_O_2_ |  | 9-Octadecenoic acid (Z)-, ethyl ester (CAS) | Antiviral, antifungal, antibacterial, antioxidant, antiinsect, anti-cancer, anti-proliferative, antifungal **[7]** |
| 20 | 39.54 | 14.37 | 17.26 | 280 | C_18_H_32_O_2_ |  | 9, 12-Octadecadienoic acid (Z, Z) - (CAS) | Decreased risk of death from all causes such as cardiovascular disease, and [coronary heart disease](https://www.sciencedirect.com/topics/pharmacology-toxicology-and-pharmaceutical-science/ischemic-heart-disease), reducing blood clots, antifungal, pesticide antiaflatoxigenic, and cytotoxic efficacy [**2, 9**] |
| 21 | 40.05 | 40.86 | 1.33 | 255 | C_16_H_33_NO |  | Hexadecanamide(CAS) | Antioxidants, hypocholesterolemic, nematicide, and pesticide [**3**] |
| 22 | 41.72 | 7.10 | 0.09 | 294 | C_19_H_22_N_2_O |  | Aspidofractinine, 3-oxo- (CAS) | - |
| 23 | 42.09 | 15.75 | 0.11 | 344 | C_18_H_16_O_7_ |  | Quercetin 7, 3', 4'-Trimethoxy | Radical-scavenging, anti-inflammatory, antibacterial, anti-insect, antiviral [**3**] |
| 24 | 42.59 | 11.67 | 0.17 | 352 | C_23_H_44_O_2_ |  | N-Propyl 11-eicosenoate | - |
| 25 | 42.97 | 43.08 | 4.93 | 281 | C_18_H_35_NO |  | 9-Octadecenamide (CAS) | Antifungal, antibacterial, larvicidal, hypolipidemic agent [**11]** |
| 26 | 43.33 | 21.71 | 0.76 | 281 | C_18_H_35_NO |  | 9-Octadecenamide, (Z)- | Antimicrobial, Anti-inflammatory, hypolipidemic agent, antimicrobial activity [**5**] |
| 27 | 44.28 | 6.16 | 1.14 | 324 | C_15_H_21_BrN_2_O |  | 10-Bromo-1,2,3,4,5,6- hexahydro-1,5-imino-9 -methoxy-3,8,11-trimethyl-3-benzazocine | [Anticancer/anti-HIV drugs](http://www.joac.info/ContentPaper/2016/2.pdf) [**1**] |
| 28 | 44 .74 | 8.94 | 0.20 | 0 | N/A |  | Hahnfett | - |
| 29 | 44.85 | 12.11 | 0.16 | 534 | C_28_H_38_O_10_ |  | 9-Desoxo-9x-hydroxy7-ketoingol 3, 8, 9, 12-tetraacetate | - |
| 30 | 45.32 | 7.93 | 0.90 | 390 | C_24_H_38_O_4_ |  | 1, 2-Benzenedicarboxylic acid, bis (2-ethylhexyl) ester (CAS) | Anti-cancer, anti-microbial, anti-fungal, anti-malarial, anti-oxidant, anti-scabies, anti-inflammatory, anti-diabetic [**2**] |
| 31 | 47.27 | 12.23 | 0.38 | 412 | C_29_H_48_O |  | Stigmasta-5, 22-dien-3- ol, (3á, 22E) - (CAS) | Antihepatotoxic, anti-inflammatory, antinociceptive, antiophidic, antioxidant; antiviral, cancer-preventive [**12**] |
| 32 | 49.25 | 53.26 | 5.16 | 337 | C_22_H_43_NO |  | 13-Docosenamide, (Z)- | Anticancer, artemicide, insecticide, antimicrobial, antioxidant scavenges activity [**5**] |
| 33 | 49.83 | 26.93 | 1.14 | 256 | C_16_H_20_N_2_O |  | 1-Propyl-2-methyl-7-me thoxy-5H, 6H-pyrido [3, 4-b] indole | Antimalarial, anticholinesterase, [antiviral, anti-inflammatory, anticancer, anti-HIV, antioxidant, antimicrobial, antitubercular, antidiabetic, activities](https://ejabf.journals.ekb.eg/article_30550_d4f0d70f306ad503ce211285e650fd5e.pdf) [**11**]. |
| 34 | 51.49 | 80.21 | 0.15 | 648 | C_44_H_56_O_4_ | - | 5, 11, 17, 23-Tetra-t-butyl -25, 26, 27, 28-tetrahydroxycalix-4-arene | - |
| 35 | 51.78 | 34.87 | 0.48 | 548 | C_35_H_48_O_3_S |  | 9(11)-Dehydroergosterol tosylate | Antifungal, chemosensitizing agent, antioxidant **[12**]. |
| 36 | 52.21 | 35.29 | 0.28 | 646 | C_29_H_2_7BrO_12_ |  | Methyl 2-(4, 8-Diacetoxy-3-brom o-6-methoxy-9, 10-diox o-9, 10-dihydroanthraqu inon-2-ylmethyl)-4-(2- methyl [1, 3] dioxolan-2- yl)-3-oxobutanoate | - |
| 37 | 52.42 | 10.65 | 0.80 | 402 | C_24_H_18_O_6_ |  | 1,1',7a,7a'-Tetrahydro6,6'-dimethoxy-1a,1a'-b i-(1aH)cyclopropa[b]na phthalene-2,2',7,7'-tetrone | - |
| 38 | 53.01 | 72.59 | 0.20 | 416 | C_32_H_32_ |  | 2, 7-Di-tert-Butyl-3, 6-diphenylbiphenylene | Fungicidal, nematicidal, larvicidal, ovicidal, insecticidal activity, antiviral, antioxidant[**13**] |
| 39 | 54.95 | 39.75 | 0.26 | 430 | C_28_H_46_O_3_ | - | Methyl 5-oxa-5,6-secocholest3-en-6-oate | - |

**References**

1. [El-Bondkly, E.A.M](https://www.scopus.com/authid/detail.uri?authorId=57218647950)., [Al Shammari, B](https://www.scopus.com/authid/detail.uri?authorId=57771577200)., [El-Gendy, M.M.A.A](https://www.scopus.com/authid/detail.uri?authorId=17345728300), [El-Shenawy, F.S](https://www.scopus.com/authid/detail.uri?authorId=57221290117).& [El-Bondkly, A.M.](https://www.scopus.com/authid/detail.uri?authorId=24340639700) Phytochemical Screening, Antifungal, and Anticancer Activities of Medicinal Plants *Thymelaea Hirsuta, Urginea Maritima*, and *Plantago Albicans*. *Gen. Rese* . 9544915 (2022).
2. Sunita, A., Ganesh ,K.& Sonam, M. Gas chromatography-mass spectroscopy analysis of root of an economically important plant, *Cenchrus ciliaris* l. from thar desert, rajasthan (INDIA). *Asi J Pharma Clin Rese*. (**10**) 9, 64-69. doi:10.22159/ajpcr.2017.v10i9.19259 (2017).
3. Kusmiati, K., Fanani, A., Nurkanto, A., Purnaningsih, I., Mamangkey, J., Ramadhani, I., Nurcahyanto, D.A., Simanjuntak, P., Afiati, F., Irawan, H., Puteri, A.L., Ewaldo, M.F. & Juanssilfero, A.B. Profile and in silico analysis of metabolite compounds of the endophytic fungus *Alternaria alternata* K-10 from *Drymoglossum piloselloides*as antioxidants and antibacterials . *Heliyon*. **10**(6) e27978 doi: 10.1016/j.heliyon.2024.e27978. PMID: 38524563; PMCID: PMC10958433 (2024).
4. El-Gendy, M.M.A.A., Yahya, S.M.M., Hamed, A.R., et al. Phylogenetic Analysis and Biological Evaluation of Marine Endophytic Fungi Derived from Red Sea Sponge *Hyrtios erectus*. *Appl. Biochem. Biotechnol*.  **185,** 755–777 <https://doi.org/10.1007/s12010-017-2679-x> (2018).
5. El-Gendy, M.M.A.A., Yahya, S.M.M., Hamed, A.R, et al. Assessment of the phylogenetic analysis and antimicrobial, antiviral, and anticancer activities of marine endophytic Streptomyces species of the soft coral *Sarcophyton convolutum*. *Int Microbiol.*  **25**,133–152 <https://doi.org/10.1007/s10123-021-00204-x> (2022).
6. [Niazi](https://www.researchgate.net/scientific-contributions/Shaik-Kalimulla-Niazi-2144422264?_tp=eyJjb250ZXh0Ijp7ImZpcnN0UGFnZSI6InB1YmxpY2F0aW9uIiwicGFnZSI6InB1YmxpY2F0aW9uIn19), S.K., [Dhanyakumara, S.B](https://www.researchgate.net/profile/Dhanyakumara-S-B).,  [Kumaraswamy](https://www.researchgate.net/scientific-contributions/Sushma-Hatti-Kumaraswamy-2249755387?_tp=eyJjb250ZXh0Ijp7ImZpcnN0UGFnZSI6InB1YmxpY2F0aW9uIiwicGFnZSI6InB1YmxpY2F0aW9uIn19), S.H.& [Bepari](https://www.researchgate.net/profile/Asmatanzeem-Bepari), A. GC-MS Based Characterization, Antibacterial, Antifungal and Anti-Oncogenic Activity of Ethyl Acetate Extract of *Aspergillus niger* Strain AK-6 Isolated from Rhizospheric Soil.  [*Molec. Biolo*](https://www.researchgate.net/journal/Current-Issues-in-Molecular-Biology-1467-3045?_tp=eyJjb250ZXh0Ijp7ImZpcnN0UGFnZSI6InB1YmxpY2F0aW9uIiwicGFnZSI6InB1YmxpY2F0aW9uIn19)*.* **45**(5), 3733-3756 DOI:[10.3390/cimb45050241](http://dx.doi.org/10.3390/cimb45050241#_blank) (2023).
7. Viswakethu, V., Ramasamy, V., Balakrishnan, P., Narayanasamy, B.& Karthic, R. Efficacy of botanical pesticides in insecticidal activity against the banana fruit scarring beetle Basilepta subcostata an In vitro analysis . [*J. Natu. Pestic. Rese*](https://www.sciencedirect.com/journal/journal-of-natural-pesticide-research). [**11**](https://www.sciencedirect.com/journal/journal-of-natural-pesticide-research/vol/11/suppl/C) , 100101 (2025).
8. Al-Marzoqi, A., Hameed, I. & Idan, S. Analysis of bioactive chemical components of two medicinal plants (*Coriandrum sativum* and *Melia azedarach*) leaves using gas chromatography-2015mass spectrometry (GC-MS). *Afri. J. BIOTE*. **14, 2812**-2830 10.5897/AJB.14956 (2015).
9. Gurunathan, A., Senguttuvan, J. & Paulsamy, S. Evaluation of Mosquito Repellent Activity of Isolated Oleic Acid, Eicosyl Ester from *Thalictrum javanicum*. *Indian. J. Pharm. Sci*. **78**(1), 103-10 doi: 10.4103/0250-474x.180259. PMID: 27168688; PMCID: PMC4852559 (2016).
10. Motleb, A.A., Aziz, M.A, et al. Chemical characterization, antimicrobial, antioxidant and larvicidal activities of certain fungal extracts. *J. Adv. Biotechno. Exp. Ther*. **5**(3), 456-472(2022).
11. Manali, D.& Goutam, C. Octadecadienoate derivatives from *Michelia champaca* seed extract as potential larvicide and pupicide against Dengue vector *Aedes albopictus*. *BMC Research Notes*. **16**. 10.1186/s13104-023-06487-9(2023).
12. Farhat, H., Urooj, F., Sohail, N., Ullah, S. & Aamer, M. Evaluation of Antimicrobial Potential of Endophytic Fungi and GCMS Metabolic Profiling of *Cephalosporium sp*., and *Fusarium moniliforme*. *J. Exp. Pathol*. **4**(1), 16-23(2023).
13. Baz, M.M., Selim, A., Radwan, I.T., Alkhaibari, A.M. & Khater, H.F. Larvicidal and adulticidal effects of some Egyptian oils against *Culex pipiens*, *Scient Rep.* **12**,4406 <https://doi.org/10.1038/s41598-022-08223-y> (2022).
